# Label‐Free and Regenerative Electrochemical Microfluidic Biosensors for Continual Monitoring of Cell Secretomes

**DOI:** 10.1002/advs.201600522

**Published:** 2017-03-06

**Authors:** Su Ryon Shin, Tugba Kilic, Yu Shrike Zhang, Huseyin Avci, Ning Hu, Duckjin Kim, Cristina Branco, Julio Aleman, Solange Massa, Antonia Silvestri, Jian Kang, Anna Desalvo, Mohammed Abdullah Hussaini, Su‐Kyoung Chae, Alessandro Polini, Nupura Bhise, Mohammad Asif Hussain, HeaYeon Lee, Mehmet R. Dokmeci, Ali Khademhosseini

**Affiliations:** ^1^ Biomaterials Innovation Research CenterDepartment of MedicineBrigham and Women's HospitalHarvard Medical SchoolBostonMA02139USA; ^2^ Harvard‐MIT Division of Health Sciences and TechnologyMassachusetts Institute of TechnologyCambridgeMA02139USA; ^3^ Izmir Katip Celebi UniversityFaculty of Engineering and ArchitectureDepartment of Biomedical Engineering35620IzmirTurkey; ^4^ Department of Metallurgical and Materials EngineeringFaculty of Engineering and ArchitectureEskisehir Osmangazi University26040EskisehirTurkey; ^5^ Biosensor National Special LaboratoryKey Laboratory of Biomedical Engineering of Ministry of EducationDepartment of Biomedical EngineeringZhejiang UniversityHangzhouZhejiang310027P. R. China; ^6^ Politecnico di TorinoDepartment of Electronics and Telecommunications (DET)Corso Duca degli Abruzzi 2410129TorinoItaly; ^7^ Programa de Doctorado en BiomedicinaUniversidad de los AndesSantiago7620001Chile; ^8^ Department of Electrical and Computer EngineeringKing Abdulaziz UniversityJeddah21589Saudi Arabia; ^9^ Department of Pharmaceutical SciencesNortheastern UniversityBostonMA02115USA; ^10^ Department of Bioindustrial TechnologiesCollege of Animal Bioscience and TechnologyKonkuk UniversityHwayang‐dong, Kwangjin‐guSeoul05029Republic of Korea; ^11^ Department of PhysicsKing Abdulaziz UniversityJeddah21569Saudi Arabia

**Keywords:** electrochemical biosensors, electrode regeneration, microfluidic, organ‐on‐a‐chip, secreted biomarkers

## Abstract

Development of an efficient sensing platform capable of continual monitoring of biomarkers is needed to assess the functionality of the in vitro organoids and to evaluate their biological responses toward pharmaceutical compounds or chemical species over extended periods of time. Here, a novel label‐free microfluidic electrochemical (EC) biosensor with a unique built‐in on‐chip regeneration capability for continual measurement of cell‐secreted soluble biomarkers from an organoid culture in a fully automated manner without attenuating the sensor sensitivity is reported. The microfluidic EC biosensors are integrated with a human liver‐on‐a‐chip platform for continual monitoring of the metabolic activity of the organoids by measuring the levels of secreted biomarkers for up to 7 d, where the metabolic activity of the organoids is altered by a systemically applied drug. The variations in the biomarker levels are successfully measured by the microfluidic regenerative EC biosensors and agree well with cellular viability and enzyme‐linked immunosorbent assay analyses, validating the accuracy of the unique sensing platform. It is believed that this versatile and robust microfluidic EC biosensor that is capable of automated and continual detection of soluble biomarkers will find widespread use for long‐term monitoring of human organoids during drug toxicity studies or efficacy assessments of in vitro platforms.

## 1 Introduction

Current paradigms for testing drug efficacy and toxicity are time‐consuming, ineffective, and expensive.^1^ One of the main reasons is that the animal models used in drug screening are often ineffective at predicting human responses to candidate drugs.^2^ In addition, ethical issues surrounding the use of animals for these studies have grown exponentially in the past few years.^3^ Therefore, there is an increasing demand for improved in vitro 3D organ models that better predict the physiological responses of the human body to novel pharmaceutical compounds, particularly with respect to tissue/organ toxicity. While conducting drug discovery studies on organ constructs it is crucial to keep track of the viability and metabolic activity of the tissue constructs/organoids. Soluble biomolecules secreted by cells can be used as indicators to assess the functionality of the in vitro organoids and evaluate their biological responses toward pharmaceutical compounds or toxic chemical species.^4^ Quantitative detection of biomarkers in complex biological media from in vitro organoids holds enormous promise in monitoring direct information regarding the status of the cells as well as the efficacy and toxicity of a drug on cells.^5^ Accurate monitoring of biological functions of cells (e.g., levels of cell‐secreted biomarkers, enzyme, etc.)^6^ requires detection and analysis of trace amounts of biomarkers of interest over an extended period of time.^7^ For example, many drugs or toxic compounds may result in chronic cellular reactions or delayed cell responses and lead to gradually changing secretion of biomarkers typically at low abundance. Therefore, the development of an efficient biosensing platform capable of accurate, continual, and long‐term monitoring of biomarkers is needed to assess the biological response of the cell or tissue construct to pharmaceutical compounds or toxic chemicals.[[qv: 4c,8]]

To address this challenge, several requirements shall be satisfied when designing robust biosensors for biomarker monitoring. First, they should be able to detect trace amounts of biomarkers (<1 ng mL^−1^) within complex biological environments such as cell culture medium, which usually contains a plethora of nonspecific proteins and interfering compounds. Second, the robust biosensor systems should be able to have continual monitoring capability every few hours or days for kinetics analysis of biomarkers over extended periods. Most of the current biosensing systems based on enzyme‐linked immunosorbent assay (ELISA),^9^ surface plasmon resonance,^10^ mass spectrometry (MS),^11^ surface enhanced Raman spectroscopy,^12^ as well as electrochemical (EC) and fluorescence‐based detection^13^ inherently suffer from surface saturation upon binding of target molecules, limiting their multiple use in accurate and continual analysis of biomarkers.^14^ To regenerate the probe, the entire interface must be reconstructed, which often requires harsh, time‐consuming stripping protocols.^15^ Moreover, potential damage incurred to the sensor surface during regeneration can also affect sensitivity.^16^ Recently, various cleaning processes have been developed to regenerate sensor surfaces while maintaining their sensitivity.[[qv: 5a,16b,17]] However, these regenerated biosensors have not been shown for their ability to continually monitor secreted biomarkers by the organoids. Ideally, the robust biosensor systems should allow for harsh and/or complicated cleaning processes to enable repeated measurements lasting from hours to days in a continual manner without the need for replacement of the biosensors. This regeneration process is critical for reusing the sensors and must be properly designed. Successful regeneration of the sensor surface should clearly restore the original sensor surface obtained after fabrication. In addition, the signal variation should be less than 5% of the initial measurements while the sensitivity of the sensor should not be significantly attenuated.^18^ Third, the sensors should be scalable to allow for simultaneous or sequential monitoring of several biomarkers. Fourth, they should be compatible with the microfluidics technology and enable convenient integration with bioreactor platforms to achieve continual and long‐term monitoring of dynamic cell or tissue behaviors.^19^ Finally, the sampling should consume minimum volume due to the typically small amount (<few mL) of the medium circulating in most microfluidic organoid platforms.

Among the many different biosensor systems currently existing, EC sensors possess unique advantages due to their excellent limit of detection (down to attomolar levels),^20^ capacity of label‐free detection, a wide linear response range, portability, suitability for long‐term monitoring, and simplicity of fabrication compared to other techniques such as MS.^21^ EC sensors can be conveniently functionalized with antibodies to achieve specific binding and detection of desired biomarkers, which potentially allows for robust surface cleaning when necessary. In addition, EC sensors are amenable to microfabrication; hence, miniaturization of EC sensors using microfabricated electrodes facilitates their integration into microfluidic platforms, allowing for suitable integration with organ‐on‐a‐chip systems. Using on‐chip valves one can further conduct sophisticated fluid manipulation to achieve repeated measurements and surface cleaning cycles in an automated manner through programmed computer controls. This feature permits labor‐free testing reducing human errors, requires low sample volumes, and allows long‐term testing. Furthermore, multiorganoid monitoring can be carried out using multiplexed sensors for high‐throughput applications, which has remained as a setback for other conventional testing techniques such as ELISA. In summary, microfluidic EC immunosensors equipped with built‐in surface regeneration capacity can enable continual measurements of secreted biomarkers from organoid platforms and allow for reliable long‐term evaluations upon drug and chemical exposures. In addition, they could have a great impact to the field since monitoring the metabolic state of cells can be a highly dynamic process. ELISA is usually done at end points and single times, which is a disadvantage since the continual changes in cellular response during testing are not captured. Therefore, the comprehensive state of the organoids can only be recognized by continual monitoring of the substrates and their metabolites but not by taking a snapshot at a single point of time.[[qv: 5c,d,22]]

In this work, we report a unique label‐free EC microfluidic immunobiosensor platform with a built‐in regeneration capability for continual monitoring of cell‐secreted biomarkers from human cell‐based liver‐on‐a‐chip. The fabrication of microfluidic valves on the same chip allowed one to carry out the regeneration and detection processes without any manual operations, which has been rarely achieved to date. The sensor utilizing EC impedance spectroscopy (EIS) measurements was adopted for label‐free detection of liver biomarkers including human albumin and glutathione‐S‐transferase‐alpha (GST‐α) to monitor hepatotoxicity. Albumin is a representative biomarker of the healthy liver tissue synthesized by the liver and GST‐α is another liver biomarker shown to increase following acute liver injury.^23^ An electrode surface cleaning process was developed and systematically optimized for gold (Au) microelectrodes to enable successful regeneration of electrode surfaces upon sensor saturation. The microfluidic EC biosensors were subsequently integrated with a primary human liver‐on‐a‐chip system where albumin and GST‐α secreted by the liver organoids were continually monitored for up to 7 d. Changes in metabolism of the liver organoids were induced by the administration of different doses of a liver‐toxic drug acetaminophen (APAP). The changes in the biomarker levels detected by the EC biosensors were compared with cellular viability and ELISA analyses to validate the accuracy of our sensing platform. We believe that this robust microfluidic EC immunobiosensor system is capable of automated and continual detection of soluble biomarkers through repeated regeneration cycles within the physiological range down to nanomolar,^23^ has not been reported to date. Our platform with carefully optimized functionalization, detection, and regeneration protocols will likely find widespread applications in cases where high‐sensitivity, streamlined detection of biochemical species is required, such as clinical diagnostics, point‐of‐care diagnosis, single cell monitoring, in addition to screening of drug toxicity, efficacy, and pharmacokinetics in organ‐on‐a‐chip systems.

## 2 Result and Discussion

### 2.1 Design and Optimization of a Label‐Free EC Sensing Method

**Figure 1:**
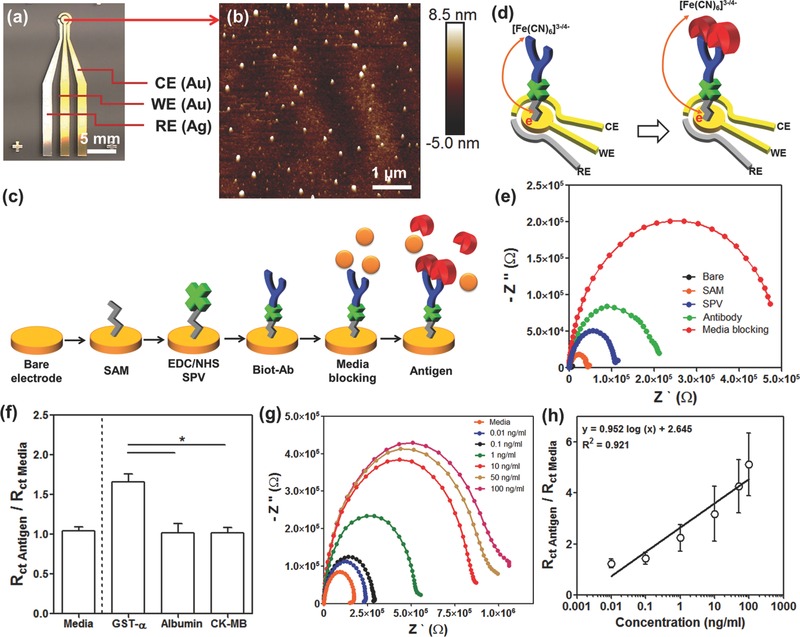
Detection principle of the label‐free EC biosensing system by using microelectrode. a) A photograph of the fabricated microelectrode having RE (Ag), WE (Au), and CE (Au). b) AFM image of the bare WE surface. c) A schematic illustration for immobilization of antibody using SPV on the surface of the microelectrodes. d) Schematic of charge transfer after antigen binding upon antibody–antigen binding for [K_3_Fe(CN)_6_]^3−/4−^ redox process. e) Nyquist plots obtained from measurements before and after the deposition of each layer (SAM, SPV, biotinylated anti‐albumin, media blocking). f) Selectivity study of albumin biosensor showing the effect of media incubation (left side from dotted line). GST‐α, albumin, and CK‐MB incubations on the GST‐α biosensor represented showing the obtained normalized *R*_ct_ values (right side from dotted line). g) Nyquist plots drawn for different standard human albumin concentrations. h) Calibration curve for human albumin plotted according to the normalized *R*_ct_ (*R*_ct_ antigen/*R*_ct_ media) values.

Microscale electrodes were designed to create an EC detection system with easy integration into microfluidic devices. The EC sensor system consisted of three electrodes, namely, a reference electrode (RE), a working electrode (WE), and a counter electrode (CE) as shown in **Figure** **1**a. It should be noted that, with the built‐in RE, we can maintain a desired and stable electrical potential between the WE and electrolyte‐ or cleaning‐solution during the impedance measurement or cleaning process, respectively. The CE and WE were made of Au whereas the RE was made of silver (Ag). Au was selected as the material for WE due to its relatively good stability, favorable electron transfer kinetics with high in‐plane conductivity, biocompatibility, and its ability to readily create covalent bonding for generating stable immobilization of receptors onto its surface. The microelectrodes were fabricated using a shadow mask and e‐beam evaporation process. The lift‐off and wet‐etching methods were not suitable for the patterning of Ag electrodes mainly due to the weak adhesion between the titanium (Ti) adhesion layer and the Ag layer which resulted in the detachment of the Ag layer during the washing steps. The washing steps are commonly used after removing the sacrificial photoresist layer using acetone or following the wet etching of the Ag layer using H_2_O_2_‐based etchant solution. Shadow mask processing can be easily utilized in microfabrication of Ag films since after peeling off the shadow mask from the wafer, the required patterns were realized on the glass substrate without the need for any wet processing. Furthermore, a palladium (Pd) barrier layer was deposited between the Au and the Ti layer and the Ag and the Ti layer, to improve the adhesion between the Ag and the Ti layer^24^ and prevent the interlayer diffusion between Ti/Au/Ag layers during the annealing process (300 °C for 6 h in a furnace).^25^ The annealing process allowed us to create robust microelectrodes resulting in minimal damage to the electrodes under harsh environments such as low and high pH solutions, corrosive solvents, and application of high electrical potentials. The microelectrode structures consisted of three layers, Ti/Pd/Au or Ti/Pd/Ag with the thicknesses of 20 nm/20 nm/500 nm as depicted in Figure S1a (Supporting Information), whereas the geometry and the dimensions of the microelectrodes are shown in Figure S1b (Supporting Information). Following fabrication, the surface of the WE was observed to be uniform and smooth (root‐mean‐square (RMS) roughness: 3.02 ± 0.45 nm) as seen in the atomic force microscopy (AFM) image (Figure 1b and Figure S1c, Supporting Information). This is crucial for improving the uniformity in immobilization of antibodies on the electrode surface, which might help improve the antigen binding capacity and result in enhanced detection sensitivity of the sensor.^26^ Furthermore, Figure S1d (Supporting Information) shows the outcome of EIS measurements obtained from a bare microelectrode which followed the expected theoretical outcome, including a semicircle plot at high frequencies and a straight line at low frequencies characteristic of the diffusion‐limited charge transfer.

As shown in Figure 1c, the surface of the microelectrodes was functionalized with antibodies specifically chosen for capture of target antigens. To create the antigen‐capture surface, the surface of the microelectrodes was first coated with a self‐assembled monolayer (SAM) by using 11‐mercaptoundecanoic acid (11‐MUA). Streptavidin (SPV) was next immobilized by covalent bonding on the SAM functionalized electrode in order to improve the alignment of antibodies. Then by using N‐ethyl‐N′‐(3‐dimethylaminopropyl) carbodiimide (EDC)/N‐hydroxysuccinimide (NHS) conjugation, a covalent bond was formed between the surface and the self‐assembled monolayer where the carboxyl‐terminated alkyl surface was converted to an active NHS ester by reacting with 11‐MUA. The EDC/NHS‐coated surface was able to react with amine‐containing SPV and create uniform patterns on the SAM. To recognize the specific target antigens, the biotinylated antibodies (biot‐Abs) were then decorated onto the SPV functionalized electrode surface via strong interaction between streptavidin and biotin.^27^ This strong interaction was able to keep the receptors on the surface of microelectrodes during fluid flow from the bioreactor. The label‐free detection mechanism of the microfluidic EC sensor is based on the change of interfacial electron‐transfer kinetics of the redox probe [Fe(CN)_6_]^4−/3−^ surrounding the electrode surface upon antibody–antigen binding (Figure 1d).^28^ In a certain concentration range the amount of antigens captured on the functionalized electrode surface using antibodies is proportional to the antigens in the solution where the amount of antigens collected on the electrode surface were monitored by measuring the impedance of the electrodes.^29^ Nyquist plots were obtained in the frequency range varying from 10^−1^ to 10^5^ Hz under a potential value of 0.10 V and modulation amplitude of 5 mV in 50 × 10^−3^ m K_3_Fe(CN)_6_. With the attachment of more antigens, the measured the electron‐transfer resistance (*R*_ct_) value of the electrode was found to increase due to shielding of the redox probe [Fe(CN)_6_]^4−/3−^ by antigens. Hence, the [Fe(CN)_6_]^4−/3−^ solution was added to the sampling medium so that we could distinguish the deposition of each coating step.

After the binding of each biomolecule layer on the electrode surface, *R*_ct_ of the electrodes was measured. In Figure 1e, a significant semicircle curve appeared in the high‐frequency region after immobilization of the SAM layers on the bare electrode. However, the signals at low frequencies representing the Warburg impedance disappeared compared to that of bare electrode (Figure S1d, Supporting Information). The diameter of the semicircle of the Nyquist plot (characterized by *R*_ct_) was found to increase from about 3 to 50 kΩ after the formation of SAM and increased further upon the deposition of SPV, biotinylated anti‐albumin, and media blocking layers due to the decreases in charge transfer efficiency of [Fe(CN)_6_]^3−/4−^ as a result of accumulated insulating layer on the electrode surface. Figure S1e (Supporting Information) shows an equivalent circuit model for obtained Nyquist curves after immobilization of the antibody layer. Here, *R*_s_, *R*_ct_, and *C*_dl_ represent Ohmic resistance of the electrolyte solution, the charge transfer resistance of the redox ([Fe(CN)_6_]^3−/4−^), and the double layer capacitance, respectively.

To improve the sensitivity of the EC biosensors, several parameters affecting the deposition process were optimized. The change in normalized *R*_ct_ values with respect to incubation time is shown in Figure S2 (Supporting Information) for different functionalization steps. After 1 h of incubation, the normalized *R*_ct_ values of different functionalization steps did not show a significant change (Figure S2, Supporting Information). Therefore, the incubation times for SAM (Figure S2a, Supporting Information), SPV (Figure S2b, Supporting Information), biotinylated anti‐albumin coatings (Figure S2c, Supporting Information), and the media blocking steps (Figure S2d, Supporting Information) were determined to be 1 h. Subsequently, the sensing performance of optimized EC biosensors was evaluated inside a complex sample solution. However, the use of complex media having high concentrations of nonspecific binding proteins that are 10^5^ times higher than those of the analytes of interest in the media, may alter the measurements and lead to incorrect results.^30^ To assess the applicability of our biosensor toward real samples, we carried out measurements using cell culture media, which usually included high concentrations of fetal bovine serum (FBS) containing around ≈99 mg mL^−1^ bovine serum albumin and other molecules but not the target antibody, that is, human serum albumin. This target‐free cell culture media was incubated with the electrodes twice after immobilization of the biotinylated albumin antibodies. The *R*_ct_ value obtained from electrodes following second media incubation displayed results that were similar to the ones obtained from the electrodes after the first media incubation step (Figure 1f and Figure S3a, Supporting Information, left side of the dotted line). These results indicated that the incoming human albumin antigens specifically bond to the human albumin antibodies even when the media included a large amount of nonspecific binding proteins such as bovine serum albumin. We then obtained the calibration curve in the detection range of 0.01–100 ng mL^−1^ with human albumin spiked to cell culture media as shown in Figure 1g,h. As the albumin concentration was increased from 0.01 to 100 ng mL^−1^ in cell culture media, the impedances between the WE and CE electrodes were found to increase as expected and an increase in *R*_ct_ values was thus observed. In addition, the EC sensor was capable of detecting albumin with a limit of detection (LOD) of 0.023 ng mL^−1^ and a sensitivity of 0.95 (log(ng mL^−1^))^−1^. Importantly, the measured LOD value was found to be lower than that reported in the previous study which used an impedance‐based biosensor (1.6 ng mL^−1^).^31^ Due to the fact that the SPV might induce the orientation of antibody on the surface of the electrode during the immobilization process, improved antigen binding capacity is anticipated comparing with randomly immobilized antibody by using glutaraldehyde as a conventional cross‐linker.^26^ Furthermore, our impedance‐based sensor showed lower LOD than amperometric biosensor (1 µg mL^−1^).^32^ Compared to amperometry requiring a wide potential range to obtain a current signal, the impedance measurement is performed in a range of frequencies, using alternating current of a very narrow range of small potentials (less than 10 mV). Therefore, the impedance is less destructive than amperometry to the measured biological interactions. This small perturbation of the electrochemical system can be perceived as stationary, where measurements with high precision are possible.^33^ Consequently, our sensor was more sensitive than those previously reported for detecting trace amounts of albumin.

To demonstrate the selectivity of our sensor, we introduced different concentrations of other biomarkers to the surface designed to capture GST‐α , which is a biomarker for damaged liver tissue. In this experiment, 1 ng mL^−1^ albumin and 1 ng mL^−1^ creatine kinase‐MB (CK‐MB) biomarkers, which can be secreted from damaged cardiac tissue, were used as the nonspecific antigens. As shown in Figure 1f (right side of the dotted line), the *R*_ct_ signal obtained from media with 1 ng mL^−1^ GST‐α was twice as high as the *R*_ct_ signals obtained from media alone. However, there were no significant differences in the *R*_ct_ values of GST‐α sensors obtained from media spiked with CK‐MB and albumin. This result should be attributed to the fact that our immunosensor only responded to the GST‐α antigen within the cell culture media. These measurements indicated that the developed EC sensor possessed high sensitivity and the selectivity and can be used for analyzing biomarkers from target tissue, and the system can be customized for the detection of other biomarkers, from various cell culture media containing large amounts of proteins with minimum sample depletion. Despite its advantages, one challenge with the EC sensor is that the electrodes tend to saturate rapidly making this a single‐use sensor. Hence, if one were to take multiple measurements or to monitor the organoids for longer durations, the sensors needed to be changed with new ones after each measurement. The saturation of the electrode surface is concentration‐ and time‐dependent. During our experiments, we observed that the EC sensor became saturated after conducting several measurements (up to ten times at 10 ng mL^−1^ albumin as antigen with incubation time of 1 h) (Figure S3b, Supporting Information), showing that the EC sensor surface covered with biotinylated antibody can capture a maximum of ≈100 ng mL^−1^ human albumin before saturation. Therefore, to carry out continual measurements of secreted biomarkers from organoid samples over longer periods a regeneration process is needed to clean the surface of the electrodes.

### 2.2 Off‐Chip Optimization of the Regeneration Process

**Figure 2:**
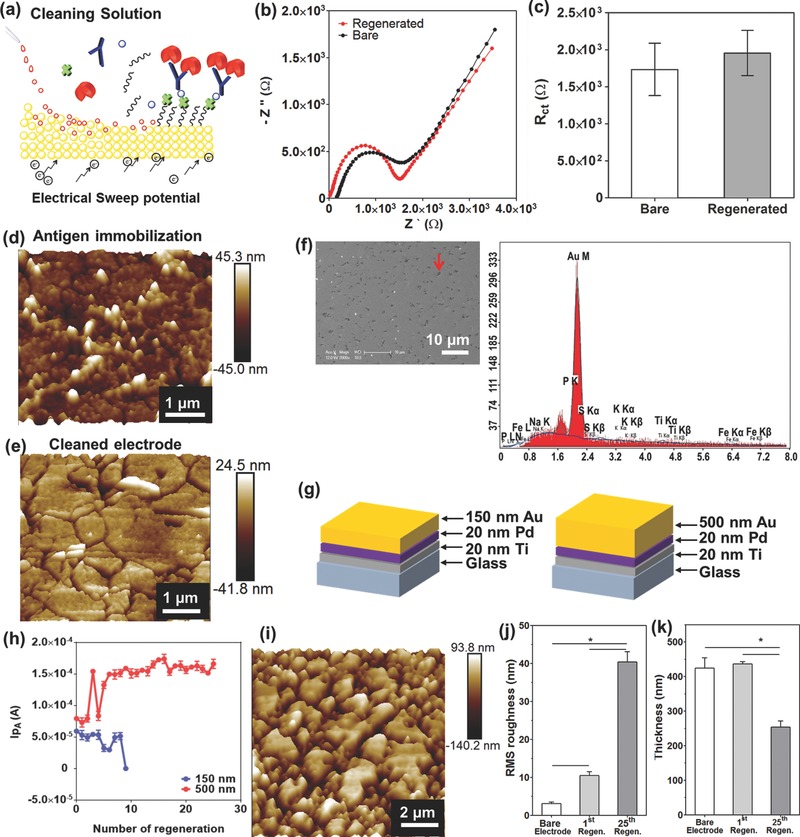
Off‐chip optimization of microelectrode regeneration. a) Schematic illustration of the microelectrode regeneration method with cleaning solution and application of electrical sweep potential. b) Nyquist plots drawn before and after regeneration of the microelectrode. c) Change in *R*_ct_ value before and after regeneration process represented by histograms with error bars (*n* = 3). AFM images of the microelectrode surface of d) after antigen immobilization and e) after regeneration. f) SEM image of regenerated microelectrode surface. EDX analysis of the spot shown by red arrow in SEM image. g) Schematic illustration of the microelectrode having different Au layer thickness, 150 and 500 nm. h)The peak current (*I*_pA_ (A)) at 0.16 V after repeated regeneration of the microelectrodes with two different Au layer thickness (150 and 500 nm) (*n* = 3). i) AFM image of 25 times regenerated microelectrode surface. j) Histograms with error bars showing the change of RMS roughness (*n* = 3) and k) thickness of bare, 1 time, and 25 times regenerated microelectrode surfaces (*n* = 3).

The EC sensor was shown to have strong binding affinities including the covalently bonded SAM, SPV‐biotin, and antibody–antigen. Therefore, the EC desorption method was selected to remove all the molecules residing on the surface of the saturated electrodes by etching thin layers of Au and carry out an electrolysis process at >±1 V (**Figure** **2**a). To create a reliable regeneration process for the EC sensor, we optimized the cleaning protocol with the goal of recovering the initial current or impedance value after removal of all immobilized layers attached to the surface. The initial values were obtained from as‐fabricated bare electrodes (Figure S4a, Supporting Information). A two‐step cleaning process was developed and optimized for the Au microelectrodes by treating their surface with H_2_SO_4_ and followed by K_3_Fe(CN)_6_ exposure under the application of electrical sweep. Sulfuric‐ and cyanide‐based cleaning solutions containing H_2_SO_4_ and K_3_Fe(CN)_6_ have been used to clean metal surfaces.^34^ The first cleaning step was optimized as follows: use of 10 × 10^−3^ m H_2_SO_4_, applied potential from 0 to 1.8 V at a 100 mV s^−1^ scan rate, and conducting an electrical sweep for five times where the process was found to be sufficient to remove the majority of antibody/antigens by breaking the thiol—Au bonds. It was demonstrated that the electrical current measured at a potential of 125 mV was found to recover ≈80% of the original current obtained after antigen binding (Figure S4, Supporting Information). However, when we further increased the number of electrical sweeps to recover ≈100% of the electrical current, the RE electrode usually underwent damage. To solve this problem, we used an additional cyanide‐based cleaning step to remove the rest of molecules by etching them from the Au layer via the formation of AuCN^−^ or Au(CN)^−^_2_ on the surface of the Au electrode in a K_3_Fe(CN)_6_ solution under an electrical sweep.^35^ Therefore, after applying the second cleaning step (50 × 10^−3^ m K_3_Fe(CN)_6_, applied potential: −1.2 to 1.2 V, scan rate: 100 mV s^−1^, number of electrical sweeps: 3), the electrical current measured from the cleaned electrodes was found to be very similar to that measured from the as‐fabricated electrodes and was significantly higher than that measured from the electrodes after antigen detection, as expected. After optimization, combination of five cycles of H_2_SO_4_ and three cycles of K_3_Fe(CN)_6_ cleaning was found to retrieve ≈100% of the signal obtained from as‐fabricated electrodes. In addition, the Nyquist plot obtained from the electrodes cleaned using the optimized cleaning process was similar to the one obtained from as‐fabricated Au electrodes (Figure 2b). No significant differences in *R*_ct_ values were observed from as‐fabricated and cleaned electrodes (Figure 2c). To validate our cleaning process, we utilized a different cleaning protocol which relied on the EC desorption of SAM in PBS using a cathodic voltage pulse.^36^ Figure S4b (Supporting Information) shows the cyclic voltammetry (CV) scans after cleaning of the electrodes in PBS using a DC pulse at −1.8 V for 30 s. This cleaning method employing PBS as cleaning solution, however, did not achieve the same value of the electrical current measured from the cleaned electrode as compared to the one obtained from as‐fabricated electrodes.

To investigate the morphological effects of each of the cleaning procedures onto the electrodes, AFM analysis was used to examine the roughness and the thickness of the cleaned microelectrodes under various conditions. The as‐fabricated Au microelectrodes displayed a homogeneous and smooth surface (RMS roughness: 3.02 ± 0.45 nm) (Figure 1b). As expected, the microelectrodes with captured antigens showed the increased surface roughness (RMS roughness: 18.49 ± 6.89 nm) since SAM/SPV/biot‐Ab/antigen layers had accumulated on the electrode surface (Figure 2d). After treating the electrodes with a solution consisting of H_2_SO_4_ and K_3_Fe(CN)_6_ under a voltage sweep, the antigens bound to the electrodes were found to be removed, resulting in a decrease in the electrode surface RMS roughness with values up to 10.46 ± 0.99 nm (Figure 2e). However, after each cleaning step, it was found that the surface roughness of the Au microelectrodes increased due to the etching of thin Au layers by the cleaning solution. This conclusion was also supported by Figure S5 (Supporting Information), which shows the results from samples that were characterized for RMS roughness and average thickness.

To determine whether the increased surface roughness after the cleaning process was caused by either re‐adsorption of the biomolecules detaching from the surface or the damage of the electrode during the etching step, SEM and the energy‐dispersive X‐ray (EDX) spectroscopy measurements were carried out. As shown in Figure 2f, the cleaned Au microelectrode contained dark spots (red arrow) on the surface which resulted in increased surface roughness. According to the EDX spectra, the composition of the spots on the regenerated electrode surface (red arrow in Figure 2f) was mainly Au, which was similar to the EDX spectra obtained from as‐fabricated electrodes (Figure S6, Supporting Information). Accordingly, the developed cleaning method successfully removed SAM/SPV/biot‐Ab/antigen molecules attached on the electrodes without re‐adsorption of the detached molecules. However, the cleaning process was found to decrease the thickness of the electrodes and hence could only be used for a finite number of times due to the etching of the Au layer during multiple cleaning steps. Accordingly, the thickness of Au layer was found to play a key role in increasing the number of electrode regeneration cycles and ensuring the high sensitivity of the sensor for long‐term and continual monitoring. To evaluate the effect of Au thickness in increasing the number of electrode regeneration steps, the microelectrodes with two different thicknesses (namely, 150 and 500 nm) were fabricated (Figure 2g). We then tested the regeneration ability of an electrode until it became unusable. We functionalized the surface of the electrodes by immobilizing SAM/SPV/biot‐Ab on the surface of the working electrodes through covalent bonding between the electrode and the SAM. The antibody immobilized microelectrode was next regenerated. We then obtained CV scans from regenerated electrodes in a 50 × 10^−3^ m K_3_Fe(CN)_6_ solution. When using a 500 nm thick microelectrode, we were able to obtain the same or similar EC oxidation signals from the redox probe (*I*_pA_) for up to 25 regeneration cycles as shown in Figure 2h. In comparison, the 150 nm thick microelectrode showed a significantly decreased *I*_pA_ after about nine regeneration cycles. Therefore, by moving to thicker Au layers (from 150 to 500 nm) in the microelectrode fabrication process, we were able to significantly increase the regeneration capability of the sensors. The thickness of electrode is the effect of the required number of regeneration steps. The electrode that was regenerated for 25 times exhibited a significant increase in the roughness and maintained half of its thickness compared to the as‐fabricated electrodes and the electrodes that were regenerated only once (Figure 2j,k). Besides, the EDX results displayed that the electrodes regenerated for 25 times seemed to have similar elemental composition of Au compared to as‐fabricated electrodes and the electrodes that were regenerated once (Figure S6, Supporting Information).

### 2.3 Design, Fabrication, and Control of the Automated Microfluidic EC Biosensor

**Figure 3:**
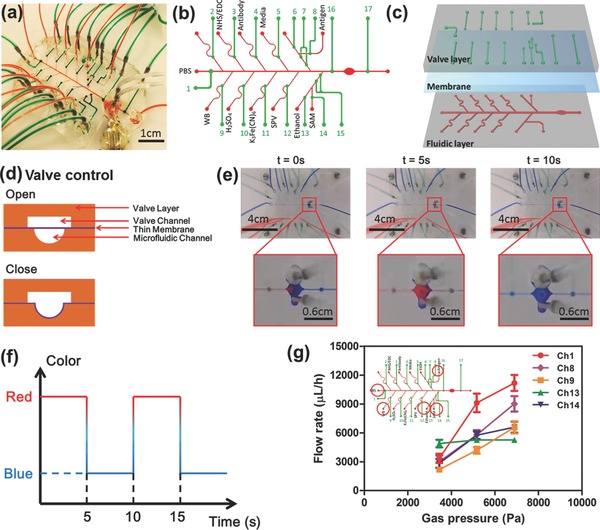
Design, fabrication, and control of the automated microfluidic EC biosensor. a) Photograph of the EC microfluidic chip bonded with microelectrode. b) Labeling of the microfluidic channels and the valves with corresponding flowing solutions for fully automated biosensing measurements. c) Three‐layered microfluidic chip consisted of microfluidic channel, thin membrane, and valve channel layer. d) Schematically represented working principle to open and close the microfluidic channel by the push‐down thin membrane according to gas pressure. e) Time‐lapsed picture of microfludic EC chip showing the color changes in the main channel for PBS and detection chamber upon opening and closing of the valves. f) Time required for changing the chemicals at the electrode area demonstrated by using food dyes. g) Measured flow rates at different channels under various gas pressures (*n* = 3).

We next developed a sensor platform with automated regeneration capability for multiple biosensor measurements. To do this, a programmable microfluidic system is required for automated sample handling, including (1) delivery of solutions at defined times to regenerate the sensor surface and will allow the sensor for long‐term (weeks) continual assays, and (2) delivery of samples from bioreactors to the sensor chip for in‐line bioassays. To achieve these requirements, the EC biosensor was integrated into a microfluidic chip, which had 13 inlet microchannels (red lines) with hemicylindrical shapes and their associated microvalves (green line) for manipulating the injection of required reagents and samples (**Figure** **3**a–c and Figure S7a, Supporting Information). The rounded design improved the closing of the microfluidic channels by blocking them with the programmed N_2_ gas‐actuated microvalves, specifically for ethanol‐based reagents such as SAM (Figure 3d).^37^ The microvalves and the reagent reservoirs were controlled by WAGO controllers with a custom‐written MATLAB code (Figure S9, Supporting Information, and Figure 3e), indicated by the time‐lapse images of the liquid flow through microfluidic channels and the detection chamber upon opening and closing of the valves. After the liquid sample reached the electrode area, it was gradually replaced by another liquid solution where the whole process took ≈5 s (Figure 3f). The flow rate in each channel was found to increase as the pressure in the channel was elevated (Figure 3g). The main channel (PBS) had the highest flow rate during the experiments due to its large and straight channel design without any curvature and/or turns. In other channels, the fluid flow rates were found to vary depending on the specific pathways. It was concluded that by changing the operating pressure, the flow rates in different channels could be tuned within a reasonable range. In addition, the bubble trap design in the chamber included pillars with 500 µm diameters which was also added to the main channel of the electrode area to prevent disruptions from the bubbles during the cleaning process, EC detection, and the flow of drugs. The diameters of the bubbles were found to dramatically decrease and they were found to disappear after about 35 s when using a chip with pillars having diameters of 500 µm (Figure S8, Supporting Information). In this microfluidic setup, the introduction of all the reagent solutions into the chip can be completely achieved in an automated manner, therefore eliminating the possibility of releasing toxic reagents including K_3_Fe(CN)_6_, SAM, and ethanol from the electrochemical sensing unit to the media in the bioreactor.^38^ Furthermore, during the impedance measurements using 50 × 10^−3^ m K_3_Fe(CN)_6_ solution, we block the flow to the detection chamber upon closure of the valves to obtain accurate impedance measurements and to reduce noise. It should be noted that, during the PBS washing step no electrical potential was applied to avoid any potential corrosion of the electrode.

### 2.4 Characterization of an Automated Microfluidic EC Chip with Regeneration Ability

**Figure 4:**
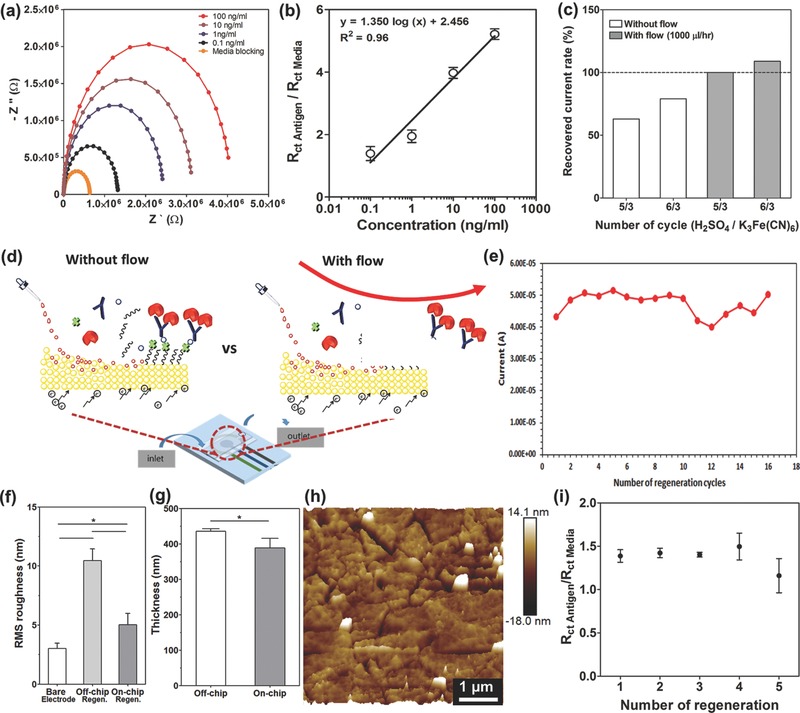
On‐chip optimization of regeneration method and sensing performance of repeated regeneration under automated manner. a) Nyquist plots for different standard human albumin concentrations. b) Calibration curve for human albumin plotted according to the normalized *R*_ct_ (*R*_ct_ antigen/*R*_ct_ media) values (*n* = 3). c) The resulting regeneration efficiencies as recovered current rate which was obtained from the normalized peak current (*I*_pA_ (A)) at 0.16 V with and without flow conditions in microfluidic chip. d) Schematics of regeneration process with and without flow conditions in microfluidic chip. e) The peak current measured at a potential of 0.16 V after repeated regeneration of the microelectrodes. Histograms showing f) the RMS roughness data (*n* = 3) and g) thickness recorded based on AFM measurements for off‐chip and on‐chip cleaning conditions (*n* = 3). h) AFM image of the microelectrode surface after on‐chip cleaning. i) Change in normalized *R*_ct_ value of antigen detection according to several number of automated regeneration (*n* = 3).

Using an on‐chip system, the calibration curve for albumin was obtained as shown in **Figure** **4**a. The fabricated EC sensor was capable of detecting albumin with a LOD of 0.09 ng mL^−1^ and a sensitivity of 1.35 (log(ng mL^−1^))^−1^. It appeared that the linear portion of the semilog calibration curve had a higher slope compared to the one obtained using the off‐chip method, demonstrating a higher sensitivity for the on‐chip immunoassay presumably due to the automated handling and manipulation of liquids in the microfluidic chip. In addition, the on‐chip measurement had a LOD of 0.09 ng mL^−1^, which was better than the one obtained from ELISA measurements (0.2 ng mL^−1^) (Table S1, Supporting Information). Overall, our microfluidic EC sensor showed better sensitivity, lower LOD, wider detection range, and required smaller sample volume compared to the conventional ELISA assay specific for albumin detection. We then optimized the parameters for cleaning of the microelectrodes using cleaning solutions with and without flow in a microfluidic chamber (Figure 4d). Initially, the microelectrode was regenerated under optimized regeneration conditions using an off‐chip system (Figure 2b), which applied an electrical sweep potential for five times in 10 × 10^−3^ m H_2_SO_4_ (applied potential: 0–1.8 V; scan rate: 100 mV s^−1^) and 50 × 10^−3^ m K_3_Fe(CN)_6_ (applied potential: −1.2 to 1.2 V; scan rate: 100 mV s^−1^), respectively. However, we found that the current obtained from the regenerated electrodes was only about 53% of the current obtained from the as fabricated electrodes (no. 1 in the table in Figure S10, Supporting Information). This result was due to the re‐adsorption of the detached molecules to the electrode surface since there was no flow of the cleaning solution in the microfluidic chamber. However, the introduction of the cleaning solution under a dynamic condition (flow rate: 1000 µL h^−1^) during the removal of the detached molecules under an electrical sweep significantly improved the regeneration process. It was found that the CV curve and *I*_pA_ value obtained from the cleaned electrode under 1000 µL h^−1^ flow rate was more similar to the one obtained from the as‐fabricated Au electrode compared to that obtained from the cleaned electrode without flow under the other conditions (Figure 4c and Figure S10, Supporting Information). Flows of cleaning solutions onto the electrodes were found to efficiently remove detached molecules from the surface of electrodes and prevent their re‐adsorption to the electrode surfaces (Figure 4d). After optimization of the cleaning process using an on‐chip system, we determined that the microelectrodes could be successfully regenerated for at least 18 times as confirmed by the generation of the CV curves that were similar to the ones obtained from the as‐fabricated electrode (Figure S11, Supporting Information). Furthermore, the current peaks (4.8 × 10^−5^ A) obtained from an electrode regenerated 18 times were similar to that obtained from the as‐fabricated electrode (4.3 × 10^−5^ A) (Figure 4e) with a maximum fluctuation of 0.9 × 10^−5^ A. To confirm the effect of flow of the cleaning solutions to the roughness of cleaned electrode, an AFM measurement was used to analyze the roughness of a microelectrode surface regenerated 18 times using cleaning solutions with and without flow. As shown in Figure 4f,g, the RMS roughness values were similar to those obtained from a bare electrode than those obtained from surface undergone an off‐chip regeneration process even though the thickness of the microelectrode which was regenerated under flow was smaller than that of the one regenerated without flow. Furthermore, AFM image (Figure 4h) displayed a smoother electrode surface after the on‐chip regeneration process compared with that of off‐chip regeneration process (Figure 2e). Consequently, we found that by supplying the cleaning solutions onto the electrodes under a continuous dynamic flow can help to efficiently remove the detached molecules from the surface of electrodes and prevent their damage during the cleaning process.

To confirm the ability to carry out continual EC detection steps using electrodes with an integrated regeneration system, the EC microfluidic chip with microelectrodes were directly connected to a WAGO controller‐driven valve controlling unit and a potentiostat (Figure 3 and Figure S9, Supporting Information) and the electrodes were regenerated up to 5 cycles (Figure 4i). After each regeneration process, 10 ng mL^−1^ of albumin concentration was measured. Relative *R*_ct_ values were determined from the Nyquist plots obtained after each regeneration cycle where 5 regeneration cycles were successfully achieved and the results were found to be consistent for up to 4 regeneration cycles. It was found that the sensitivity decrease after 4 times of regenerated microelectrode is likely due to the increased roughness of microelectrode surface, while the reason for the roughness change should be attributed to the etching of Au due to potential sweep and chemical treatment. However, in Figures 2f and 4e and Figure S11 (Supporting Information), the peak potential and current of the redox probe [Fe(CN)_6_]^4−/3−^ surrounding the electrode surface did not significantly change upon multiple regeneration processes, indicating that the microelectrode set after these many regeneration cycles had the possibility to detect an antigen. To this end, it could become a problem if we want to measure a very low amount of antigen concentration. However, it is still possible for the regenerated sensor, even after 4 cycles, to detect the increase and decrease in the level of antigen using predetermined calibration curves, although at the expense of decreased sensitivity. Based on the working mode of the microfluidic chip for each cycle, the electrodes were exposed to chemical flows ≈4 h. This length of time most probably caused more etching of thin layers of Au than expected. In addition, the current EC microfluidic chip design such as the bonding process and the durability of the membrane, and other unoptimized factors could be other limitations preventing the achievement of higher number of regeneration steps.

### 2.5 Continual Monitoring of Cell‐Secreted Biomarkers from Bioreactor Samples

**Figure 5:**
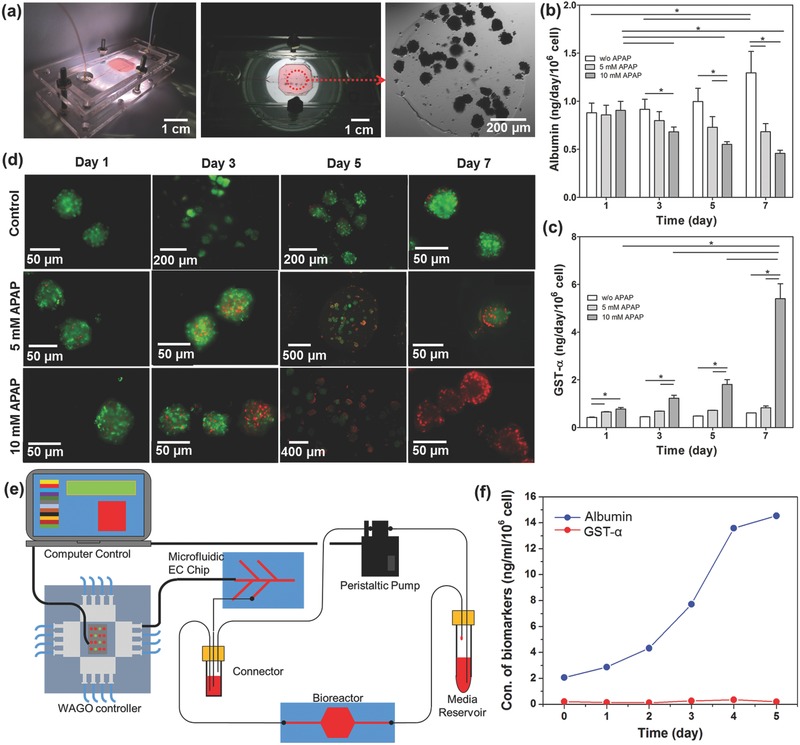
On‐chip detection of biomarkers secretion from primary hepatocyte bioreactor construct upon drug treatment. a) Sealed and primary hepatocyte spheroid patterned bioreactor with magnified view of incubation chamber and microscope image of a printed spheroid. b) Continual EC measurements of albumin (*n* = 3) and c) GST‐α production rate in primary hepatocyte bioreactor with control, 5 × 10^−3^ and 10 × 10^−3^ m APAP exposure conditions (*n* = 3). d) Live/dead staining for control, 5 × 10^−3^ m APAP exposed and 10 × 10^−3^ m APAP exposed primary hepatocyte spheroids in bioreactor at days 1, 3, 5, and 7. e) Schematic diagram of the microfluidic EC biosensing system integrated with organ‐on‐a‐chip for continual monitoring of a target biomarker by automated manner. f) Automatic continual EC measurements of albumin and GST‐α production rate in primary hepatocyte bioreactor without drug treatment.

We next analyzed the performance of our microfluidic EC biosensor to monitor biomarkers secreted by human liver organoids hosted inside microfluidic liver bioreactor. In our previous study, microfluidic‐based bioreactors were developed using advanced platforms that recapitulated the biology and physiology of human organs on an integrated microfluidic circuitry for drug and toxin screening.^39^ To fabricate the human liver bioreactor, human primary hepatocyte spheroids‐laden gelatin methacryloyl (GelMA) prepolymer solutions were bioprinted into a microfluidic bioreactor with ≈9 × 10^5^ cells in microdot‐array form (diameter: ≈600 µm) (**Figure** **5**a and Figure S12, Supporting Information). To conduct hepatotoxicity assessment studies, we used acetaminophen (APAP) as a model drug and evaluate the response of the platform against this drug. It is well known that exposure of hepatocytes to APAP can alter protein secretion rates which will be mediated by metabolic activities.^40^ In this study, 5 × 10^−3^ and 10 × 10^−3^ m APAP concentrations were utilized to induce hepatotoxicity as per the optimized dose reported in our previous study and other published literature.[[qv: 39d,41]] To monitor albumin and GST‐α levels, sample solutions were collected from the bioreactor and then delivered into one of solution reservoirs that was connected with the sampling microchannel on the EC microfluidic chip by manual operation. For the EC microfluidic chip, automatically controlled valves and microfluidic channels were programmed to allow the solution entry at the predefined time points in conjunction with the measurements obtained using the potentiostat. The secretion rate of albumin was found to increase from day 1 through day 7 for the bioreactor without APAP treatment, as expected (Figure 5b). However, when the cells were exposed to media containing 5 × 10^−3^ and 10 × 10^−3^ m APAP, the production rates of albumin from day 1 through day 7 were found to statistically decrease to more than half of its initial value. To indicate the changes in the level of secreted GST‐α, the EC calibration curve for GST‐α is shown in Figure S13 (Supporting Information) and the GST‐α biosensor was found to detect up to 50 ng mL^−1^ with a LOD of 0.01 ng mL^−1^. Therefore, we found that the developed GST‐α biosensor can detect GST‐α secretion within the physiological range which is in nanomolar concentration.^23^ When the cells were treated with 5 × 10^−3^ m APAP, the production rates of GST‐α obtained from day 1 through day 7 were found to be the same with the values obtained from the control group (Figure 5c). However, for the organoids treated with 10 × 10^−3^ m APAP, the production of GST‐α was found to significantly increase at day 1 and had finally increased by ten times at day 7 in comparison with the control sample. The albumin concentration was found to decrease whereas the GST‐α concentration was found to increase after the liver organoids were exposed to APAP.

To demonstrate the ability of the EC biosensor to monitor cell‐secreted biomarkers from a microfluidic bioreactor, the data obtained from the EC biosensor were compared with the data obtained from ELISA measurements. This comparison revealed that the change in the levels of the two biomarkers obtained by the EC biosensor and the ELISA data agreed well with each other (Figure S16, Supporting Information). To obtain a qualitative comparison between the measured values of cell‐secreted biomarkers and the viability of spheroids, we further performed a cell viability assay after the cells were exposed to different APAP concentrations. As shown in Figure 5d, the control (no APAP) showed a high viability until the end of day 7. When the cells inside the bioreactors were exposed to APAP, the viability of the cells was found to decrease with increasing APAP dose and the incubation time. For the bioreactors treated with 5 × 10^−3^ m APAP, many live cells were still found inside the bioreactors at the end of 7 d, indicating partial cytotoxicity of the drug at such an elevated concentration of APAP. However, 10 × 10^−3^ m APAP exposure significantly decreased the cell viability after day 7. These results correlated well with results obtained from the literature and our previously published study.^42^ Remarkably, our data showed that the trend in cell viability at each day was consistent with the variation of albumin and GST‐α production rates at the same time points. This observation was also in agreement with hepatotoxicity studies reporting APAPs impact on cell viability and the biomarker secretion rates.[[qv: 39d,43]] Such accurate measurement proved the reliability of the on‐chip immunoassay for detection of the target biomarkers using our EC biosensors.

To confirm the performance of the EC biosensors with an electrode regeneration capability in continually monitoring cell‐secreted biomarkers in a microfluidic bioreactor, an automated integrated system was assembled to achieve long‐term, on‐line monitoring of the changes in the albumin and GST‐α levels without APAP treatment (Figure 5e). The EC sensing platform was directly connected to the bioreactor hosting the human primary hepatocyte‐laden liver organoids. After sensing of biomarkers from the bioreactor samples at day 0, the microelectrode surface was cleaned using the optimized cleaning protocol. All the necessary individual biomolecules including SAM, EDC/NHS, SPV, biot‐Ab, and media were sequentially injected into the microchamber to refunctionalize the cleaned microelectrodes. Based on the obtained standard curve, the concentrations of bioreactor samples at 1, 2, 3, 4, and 5 d were calculated (Figure 5f). The secretion rate of albumin was found to increase from day 1 through day 5 for the samples collected from the bioreactors. However, the production rates of GST‐α obtained from day 1 through day 7 was found to be the similar with the values obtained from day 0. Therefore, our automated and integrated system was able to achieve long‐term and on‐line monitoring with multiple biomarkers using the reusable microelectrode. Furthermore, our unique sensor platform prevented variability due to human errors, unlike most of EC‐based antigen sensors which are operated manually or semiautomatically.^44^ However, these methods are relatively complicated due to sophisticated protocols, and are time‐consuming due to the longer incubation times required for the measurements. In particular, the total time required for a complete detection and regeneration cycle is ≈4 h, including the functionalization of the microelectrode (174 min), the detection of antigen (58 min), and subsequent regeneration cycle (18 min). However, after functionalization each sensor can be used for multiple detections before reaching saturation (e.g., around ten times upon multiple detections of 10 ng mL^−1^ human albumin, Figure S3b, Supporting Information), allowing for more frequent measurements; moreover, multiple sensing units can be integrated to enable alternative usage when even shorter intervals are necessary for antigen detection. Therefore, our automatic microfluidic system can efficiently improve the experiments in the following aspects: (1) fully automated operation of electrode functionalization, detection, and regeneration; (2) label‐free antigen detection process that requires minimum medium depletion; (3) regenerative capability of the electrode surface upon saturation with captured antigens; and (4) cost‐effectiveness due to the use of the miniaturized electrodes and microfluidic platform. All advantages of this automatic microfluidic EC biosensing module are superior to existing sensing systems in performing long‐term and continual monitoring of biomarkers.

## 3 Conclusion

In this paper, we have developed a reusable label‐free microfluidics EC biosensor and integrated this system with a human organoids system. The objective was to design a system for on‐line and long‐term detection of cell‐secreted biomarkers. The impedance‐based biosensor was capable of detecting albumin and GST‐α with an LOD of 0.023 and 0.01 ng mL^−1^ from complex biological environments such as cell culture medium which usually contains a plethora of nonspecific proteins and interfering compounds but trace amounts of biomarkers of interest. In addition, we have successfully optimized the electrode thicknesses and implemented the off‐chip detection process into an on‐chip microfluidic detection process with regeneration for continual monitoring of biomarkers secreted from organoids which are exposed to different concentrations of drugs under perfusion flow. The optimized cleaning strategy developed for the regeneration process enabled long‐term monitoring without any significant loss in sensor sensitivity for up to 25 regenerations. This microfluidic‐integrated label‐free EC biosensor with an on‐chip built‐in regeneration capability was successfully controlled in a fully automated manner. Moreover, the real media samples from a human liver‐on‐a‐chip bioreactor were measured using this platform, which showed similar results with those obtained from ELISA tests before and after APAP treatment. This work provided a novel and unique universal strategy for the construction of automated microfluidic based sensors for continual monitoring of the specific biomarkers involved in monitoring of cells for drug toxicity.

## 4 Experimental Section

All experimental methods are described in the Supporting Information.

## Supporting information

As a service to our authors and readers, this journal provides supporting information supplied by the authors. Such materials are peer reviewed and may be re‐organized for online delivery, but are not copy‐edited or typeset. Technical support issues arising from supporting information (other than missing files) should be addressed to the authors.

Supplementary
