# Correction to “Label‐Free and Regenerative Electrochemical Microfluidic Biosensors for Continual Monitoring of Cell Secretomes”

**DOI:** 10.1002/advs.202511422

**Published:** 2025-07-22

S. R. Shin, T. Kilic, Y. S. Zhang, H. Avci, N. Hu, D. Kim, C. Branco, J. Aleman, S. Massa, A. Silvestri, J. Kang, A. Desalvo, M. A. Hussaini, S.‐K. Chae, A. Polini, N. Bhise, M. Asif Hussain, H. Y. Lee, M. R. Dokmeci, and A. Khademhosseini, “Label‐Free and Regenerative Electrochemical Microfluidic Biosensors for Continual Monitoring of Cell Secretomes,” *Advanced Science* 4, no. 5 (2017): 1600522, https://doi.org/10.1002/advs.201600522.

Concerns were raised by a third party regarding overlapping image panels between the EDX spectra of **Figure 2**f and Figure S6d (Supporting Information), yet the spots they are reporting on are not the same.

The authors explained that the low‐magnification SEM image in Figure 2f shows the overall morphology of the electrode, and the red arrow only representatively marked one black spot, the EDX data does not belong to it. The actual spot from which the EDX data was obtained is indicated as Spot 2 in the SEM image of Figure S6c (Supporting Information), with the same EDX data shown again in Figure S6d (Supporting Information). To provide further clarification for the readers, the authors have corrected Figure 2f by removing the red arrow and revising the figure caption.

The corrected Figure 2 and the revised figure caption are below:


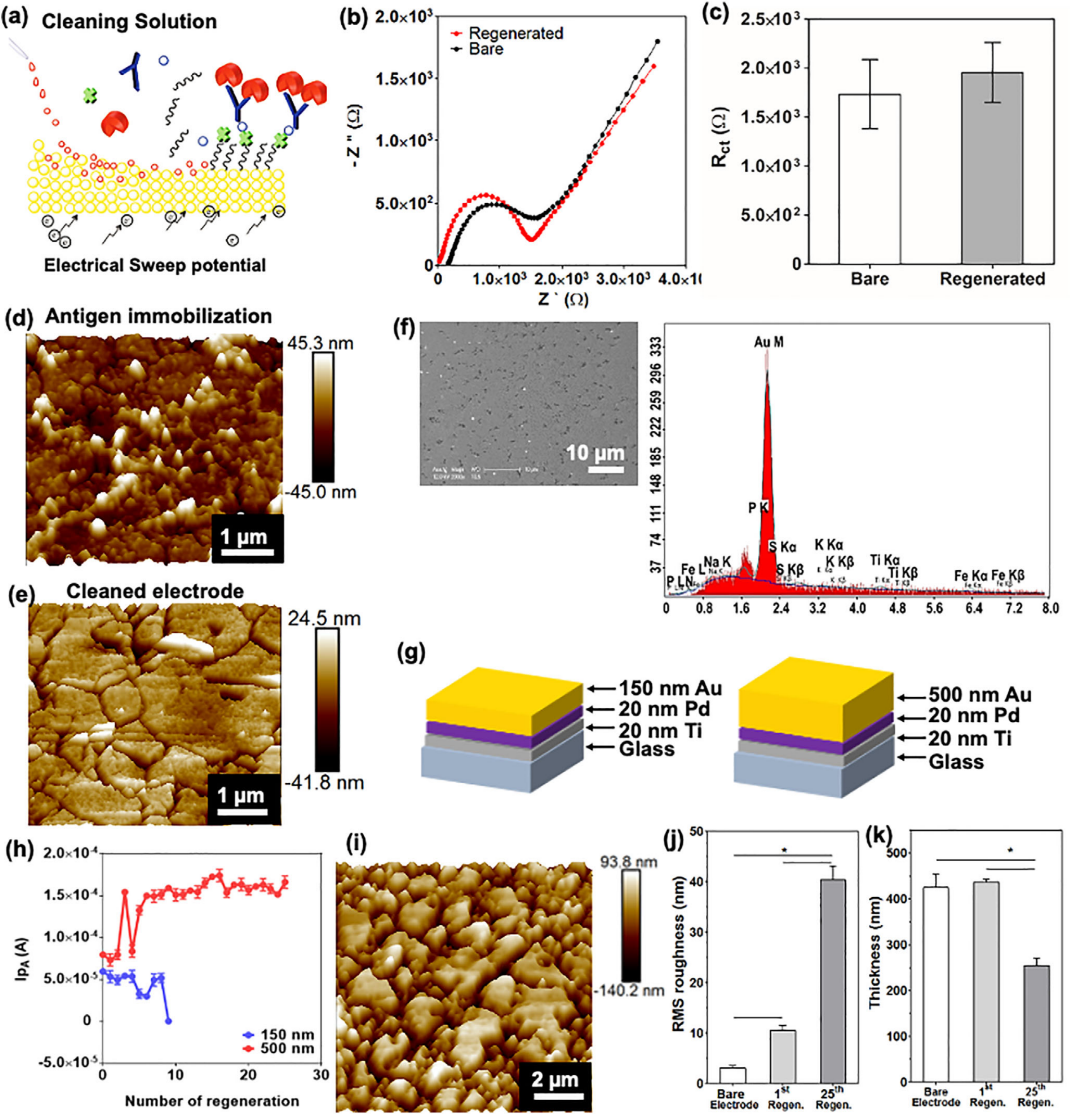


Figure 2. Off‐chip optimization of microelectrode regeneration. a) Schematic illustration of the microelectrode regeneration method with cleaning solution and application of electrical sweep potential. b) Nyquist plots drawn before and after regeneration of the microelectrode. c) Change in *R*_ct_ value before and after regeneration process represented by histograms with error bars (*n* = 3). AFM images of the microelectrode surface of d) after antigen immobilization and e) after regeneration. f) Representative SEM image of regenerated microelectrode surface to show the overall morphology of the electrode. EDX analysis of one of the black spots. The corresponding spot is indicated by a white arrow in the SEM image of Figure S6c (Supporting Information). g) Schematic illustration of the microelectrode having different Au layer thicknesses, 150 and 500 nm. h) The peak current (*I*_pA_ (A)) at 0.16 V after repeated regeneration of the microelectrodes with two different Au layer thicknesses (150 and 500 nm) (*n* = 3). i) AFM image of a 25 times regenerated microelectrode surface. j) Histograms with error bars showing the change of RMS roughness (*n* = 3) and k) thickness of bare, 1 time, and 25 times regenerated microelectrode surfaces (*n* = 3).

