# Supplementary material for: Label‐Free and Regenerative Electrochemical Microfluidic Biosensors for Continual Monitoring of Cell Secretomes
Source: Adv Sci (Weinh). 2017 Mar 6;4(5):1600522. doi: 10.1002/advs.201600522 (PMC5441508; doi:10.1002/advs.201600522)
Supplement: Supplementary file 1 — Supplementary [file ADVS-4-na-s001.pdf]

Copyright WILEY-VCH Verlag GmbH & Co. KGaA, 69469 Weinheim, Germany, 2017.

ADVANCED SCIENCE
Open Access

Supporting Information

for *Adv. Sci.,* DOI: 10.1002/advs.201600522

Label-Free and Regenerative Electrochemical Microfluidic Biosensors for Continual Monitoring of Cell Secretomes

Su Ryon Shin, Tugba Kilic, Yu Shrike Zhang, Huseyin Avci, Ning Hu, Duckjin Kim, Cristina Branco, Julio Aleman, Solange Massa, Antonia Silvestri, Jian Kang, Anna Desalvo, Mohammed Abdullah Hussaini, Su-Kyoung Chae, Alessandro Polini, Nupura Bhise, Mohammad Asif Hussain, HeaYeon Lee, Mehmet R. Dokmeci,* and Ali Khademhosseini**

Copyright WILEY-VCH Verlag GmbH & Co. KGaA, 69469 Weinheim, Germany, 2016.

Supporting Information

Label-free and Regenerative Electrochemical Microfluidic Biosensors for Continual Monitoring of Cell Secretomes

Su Ryon Shin[1,], Tugba Kilic[1], Yu Shrike Zhang[1], Huseyin Avci, Ning Hu, Duckjin Kim, Cristina Branco, Julio Aleman, Solange Massa, Antonia Silvestri, Jian Kang, Anna Desalvo, Mohammed Abdullah Hussaini, Su-Kyoung Chae, Alessandro Polini, Nupura Bhise, Mohammad Asif Hussain, HeaYeon Lee, Mehmet R. Dokmeci*, Ali Khademhosseini**

1. Experimental and Methods

1.1. Fabrication of microelectrode: The designed microelectrode for long-term monitoring of cell-secreted biomarkers was three-electrodes configuration: Ag reference electrode (RE), Au counter electrode (CE), and Au working electrode (WE). After cleaning of square glass substrates (25mm×25mm) by oxygen plasma, shadow mask process was used in order to manufacture the microelectrodes (Fig. S1). In this process, metal layers were selectively deposited over a shadow mask which has apertures in metal film of 0.25 mm thickness. In this process, the first shadow mask for WE and CE was attached to cleaned glass substrate. The 20 nm- thick Ti, 20 nm- thick Pd, and 500 nm-thick Au was deposited on the glass by using e-beam evaporation. Also, the Au electrodes were not patterned with a passivation layer. Next, the second shadow mask for RE was attached to the Au deposited glass wafer. The alignment is achieved by using alignment keys. 20 nm-thick Ti, 20 nm- thick Pd, and 500 nm-thick Ag was then deposited. After peeling off the shadow mask from the wafer, the required patterns were realized without the need for any wet processing.

1.2 Functionalization of microelectrode bonded PDMS chip: The biotinylated anti-albumin and anti-GST-α were obtained from ELISA kits which was purchased from Abcam (USA) and MyBiosource (USA), respectively. The biotinylated antibodies (biot-Ab) of interested biomarker were immobilized to the surface of microelectrode as detailed below. After two-step EC cleaning, the microelectrode was rinsed by anhydrous ethanol and 11-mercaptoundodecanoicacid (MUA, 10 mM prepared in anhydrous ethanol, Sigma). After one hour incubation of MUA, rinsing with ethanol and DPBS to remove unbounded thiols, the carboxylicacid terminated alkyl surface was converted to an active NHS ester via 7 min washing and 30 minute incubation of freshly prepared 1:1 mixture of 50 mM N-Hydroxysuccinimide (NHS, Sigma, in 50 mM citric buffer solution of pH : 4.5) and 50 mM 1-ethyl-3-(3-(dimethylamino)-propyl) carbodiimide (EDC, Sigma, in 50 mM citric buffer solution of pH : 4.5). The surface then quickly rinsed with streptavidine (SPV, Sigma, 10 μM in DI water) solution for 7 min and incubated for 1 hour. Unbounded SPV molecules were removed via 7 min washing by washing buffer (provided by ELISA kits). The biot-Ab were immobilized to the surface via SPB-Biotin interaction upon 7-minute washing of biot-Ab (1x dilution in dilution buffer, Abcam) prior to one-hour incubation. Having rinsed the biot-Ab modified microelectrode surface by the washing buffer, the surface of microelectrode was blocked via biomarker-free cell culture media for 1 hour to prevent non-specific binding of proteins.

1.3. EC detection of albumin and GST-α: Varying concentrations of human albumin and GST-α were analyzed via EC impedance spectroscopy [1] performed on a CHI 660E EC workstation (Shanghai CH Instruments Co., China) with three-electrode system fabricated on microelectrode; Au working electrode, Ag reference electrode and Au counter electrode. EIS measurements were conducted in the frequency range varying from 10^{-1} Hz to 10^{5} Hz under a potential value of 0.10

V and modulation amplitude of 5 mV in 50 mM $K_3Fe(CN)_6$. The charge transfer resistance (R_{ct}) of samples were determined via fitting of the raw data by CHI software based on the equiavalent circuit composed of the circuit elements; R_{ct}, solution resistance R_s, double-layer capacitance, C_{dl}, and Warburg impedance, W. All impedance data collected for various conditions were fitted using the same model (Figure S1(e)) and had goodness of fit values within the range of 10^{-6} and the errors associated with the fitting parameters were 2%–6%. The limit of detection (LOD) value was calculated by three times the standard deviation of the media signal.

1.4. Regeneration of microelectrode: In order to achieve the long-term monitoring, the saturated microelectrode surface was regenerated where all molecules have been removed *via* two-step regeneration procedure under flow conditions: (1) 5 segments cyclic voltammetry (CV) scan of H_2SO_4 cleaning with sweep range of 0.0-1.8 V, scan rate of 0.1 V/s and sensitivity of $1.0x10^{-2}$ A/V at line frequency of 60 Hz. (2) 3 segments CV scan of $K_3Fe(CN)_6$ cleaning via with sweep range of 1.2-(-1.2) V, scan rate of 0.2 V/s and sensitivity of $1.0x10^{-4}$ at line frequency of 60 Hz. Between consecutive steps, a washing step via DPBS was performed to remove the remnant from previous washing. After this regeneration procedure, the surface characteristics of microelectrode were tested via CV and EIS to compare with blank electrode.

1.5. Setting up a microfluidic chip: The valves controlling the microfluidic channels were first checked via flow of food dyes at 10 psi. Then all the tubing was inserted into the microfluidic channels and filled with Dulbecco's Phosphate Buffered Saline (DPBS) manually from the main outlet. The channel tubing connected to related solution reservoirs and microfluidic chip was flushed with DPBS for 7 min. Finally, the microelectrode was regenerated via two-step cleaning procedure: (1) 7 min H_2SO_4 flow, 5 segments CV with a sweep range of 0.0 V - 1.8 V, scan rate of 0.1V/s and sensitivity of 1.0 e^{-2} A/V at a line frequency of 60Hz. (2) 7 min $K_3Fe(CN)_6$ flow, 3

segments CV with a sweep range of -0.6V-0.6 V, scan rate of 0.075 V/s and sensitivity of 1.0 x10^{-2} A/V at a line frequency of 60 Hz. DPBS washing for 7 min was performed after each cleaning step.

1.6. Characterization of microelectrode surface: To characterize the surface of micro-fabricated Au electrode before and after modification via proteins and also two-step regeneration, Atomic force microscopy (AFM) and Energy Dispersive X-ray spectroscopy (EDX) have been done. AFM was performed by using Bruker Dimension Icon AFM (Bruker Corporation, Germany). The images were analyzed using the offline AFM software (NanoScope Analysis, version 1.5) in tapping-mode in air at room temperature.

1.7. Spheroid Formation and encapsulation: Primary human hepatocytes (Triangle Research Labs, Lonza, USA) were thawed (8x10^{6} cells/ml) and placed in a PDMS 200 μm multiwell mold for spheroid formation as previously described [2]. A spheroid formation medium was used for spheroid culture and refreshed each day for 5 days. The medium consisted of Hepatocyte Basal Media (Lonza), 20% Heat Inactivated Fetal Bovine Serum (Gibco) and 1μg/ml Rat Tail Collagen (Gibco). Spheroids were collected on 5th day and blended into a hydrogel solution containing 10% (w/v) gelatin methacryloyl (GelMA) and 0.5% (w/v) 2-Hydroxy-4'-(2-hydroxyethoxy)-2-methylpropiophenone as photoinitiator (PI).

1.8. Bioreactor construction, bioprinting and drug treatment: The bioreactor was assembled as formerly described [3]. A 20 dot microarray of GelMA hydrogel embedded spheroid droplets were printed by a NovoGen MMX bioprinter (CA, US). Each dot contained average 18 spheroids. The dots were printed on a tetramethylsilane (TMSPMA) treated glass and exposed under UV light exposure (850mV at 8.5 cm) for 18 s, and then GelMA dots were successfully crosslinked and attached onto the culture glass in the bioreactor. Subsequently the bioreactor was closed and

perfused with a peristaltic pump. Medium was pumped from a reservoir, passed through the bioreactor and collected on days 1, 3, 5 and 7 for ELISA and electrochemical (EC) sensing, respectively. APAP was dissolved in cell culture media and was continuously administrated as described in our previous work [4]. APAP doses of 5 mM and 10 mM were used in different bioreactors for drug toxicity evaluation on 1, 3, 5 and 7 days.

1.9. Cell counts: To count the total number of cells in the printed sample, the GelMA hydrogel embedded spheroids were treated by 1.0 mg/ml collagenase II solution at 37 ^{o}C for 10 min until the GelMA hydrogel fully degraded. We then added 1X trypsin solution for 5 min at 37 ^{o}C to obtain the single cell suspension. The obtained cell suspension was washed by the fresh culture media and then the cells were counted with a hemocytometer.

1.10. Live and dead assay and microscopy: LIVE/DEAD® viability (Life Technologies) assays were performed to assess viability on the control and APAP treated bioreactors on 1, 3, 5 and 7 days. Fluorescent images were acquired by Zeiss Axio Observer D1 Fluorescence Microscope (Carl Zeiss, Germany) was used for acquisition. Images were analyzed by ImageJ analysis software[5].

1.11. Automatic control system: The automatic control system was performed by the controller WAGO 750-881 that managed 4 solenoid valves FESTO 197020 through the 4-channel digital output module 24V DC WAGO 750-504. In order to control the microfluidic platform, 30 solenoids valves were needed. 8 modules FESTO and 8 controllers in parallel were employed. 17 solenoids are used for the valve control and 13 are used for injecting the liquids into microfluidic channels. A personal computer linked to the Fieldbus controller through MATLAB (Mathworks) programming functions performed basic valve control using WAGO devices and EC measurements in connection with CHI workstation: the system can start/stop to flow liquid

solution in different channels and open/close different microfluidic valves, start/stop EC measurements for CV and EIS. Solutions were placed in the reservoirs connected to the FESTO valves and the inlets of platform. Microfluidic valves were actuated directly from FESTO valves.

1.12. Statistical analysis: To analyze statistical significance, we used a one-way ANOVA where appropriate (GraphPad Prism 6.0, GraphPad Software). Error bar represents the mean ± standard deviation (SD) of measurements performed on each sample groups. To determine whether a significant difference exists between specific treatments, we used Tukey's multiple comparison tests ($p<0.05$).

2. Supporting Table and Figures

Table S1: Comparison of sensitivity, accuracy, LOD, accuracy, detection ranges and sample volume of the regenerative microfluidic EC sensor and and the ELISA assay for albumin detection.

	Sensitivity	LOD (ng/ml)	Accuracy (inter-assay, %)*	Detection range (ng/ml)	Sample volume (µL)
ELISA	1.5 ng/ml^{-1}	0.2	8.7	3.125 - 200	50
Regenerative microfluidic EC sensor (Albumin biosensor)	1.35 $log(ng/ml)^{-1}$	0.09	9.6	0.1 - 100	7

* Average value of 4 different concentration.

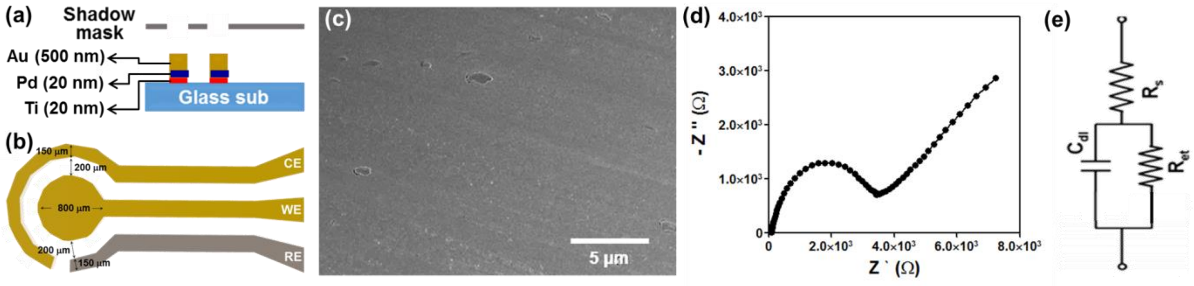

Figure S1. (a) Schematic representation of photolithographic process performed for microelectrode fabrication (b) Geometry and dimensions of the microfabricated electrode. (c) SEM image of WE surface. (d) Nyquist plots obtained from bare electrode. (e) Equivalent circuit Rs(Qdl[RetW]) used to fit the frequency scans along with label-free biosensor.

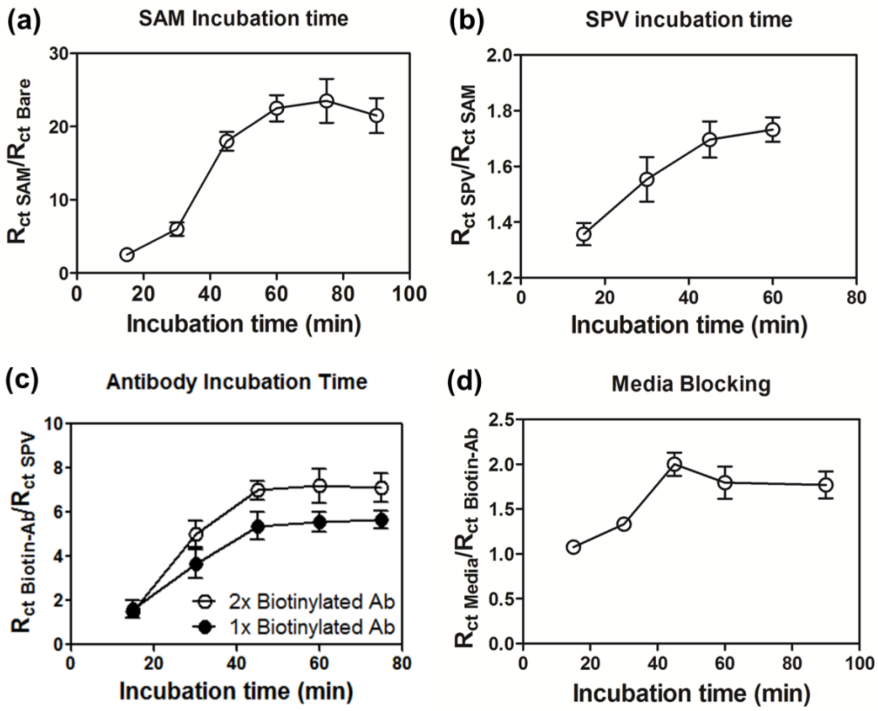

Figure S2. Optimization studies for EC sensor (n=3); (a) SAM incubation time study. (b) SPV incubation time study. (c) Biotinylated anti-albumin incubation time study. (d) Media blocking time study.

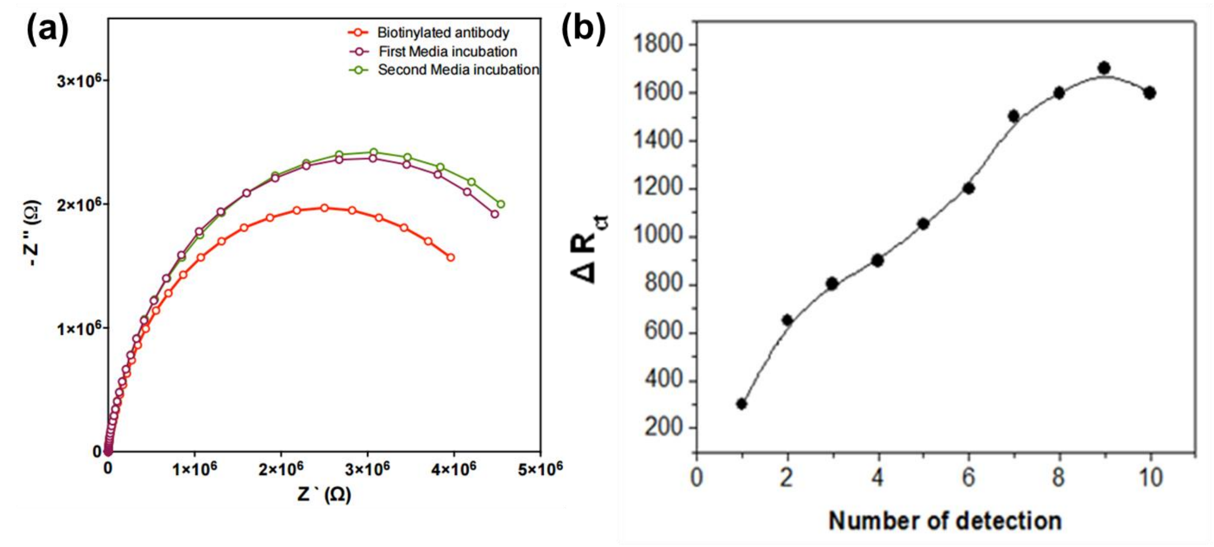

Figure S3. (a) Nyquist plots obtained from GST-α biosensors after first and second rounds of media incubation. (b) Change in Δ R_{ct} ($R_{ctSample}$ - $R_{ctMedia\ blocking}$) value upon multiple times detection of 10 ng/ml human albumin.

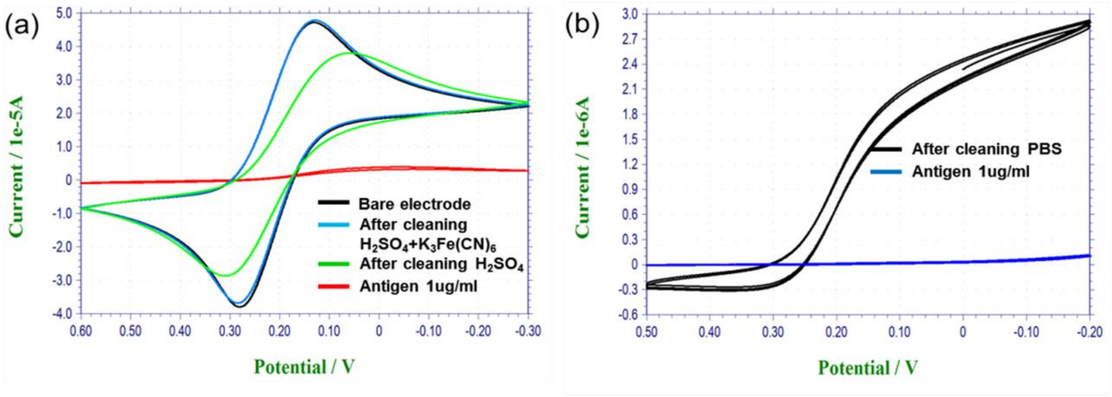

Cleaning solution	Method	Recovered current rate (at 125mV)
(1) H_2SO_4	CV sweep (0V – 1.8V)	79.93 %
(2) H_2SO_4+ $K_3Fe(CN)_6$	CV Sweep (0V – 1.8V), CV Sweep (-1.2V to +1.2V)	~100 %
(3) PBS	DC -1.8V for 30 sec	Cannot find peak current

Figure S4. (a) CVs of bare, human albumin antigen immobilized and regenerated electrode at various cleaning conditions. (b) CVs of at human albumin antigen immobilized electrode before and after cleaning by PBS and applied DC potential -1.8V.

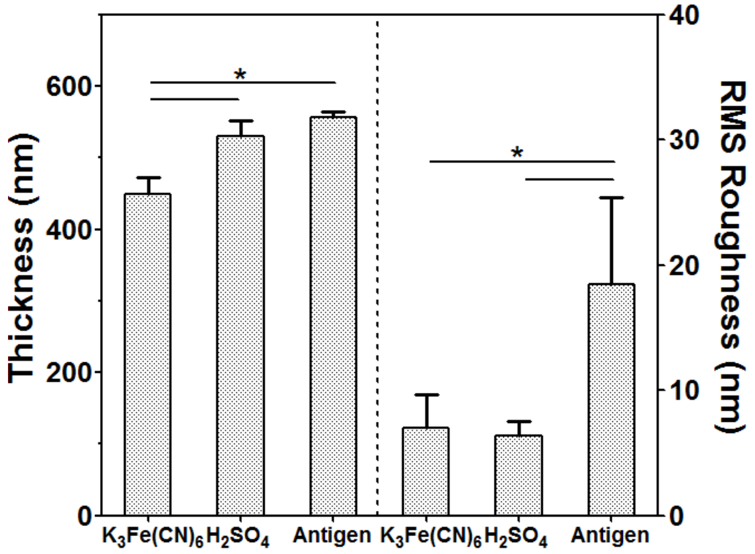

Figure S5. The average thickness and RMS roughness of the Au microelectrode determined according to AFM images for different electrode samples (n=3).

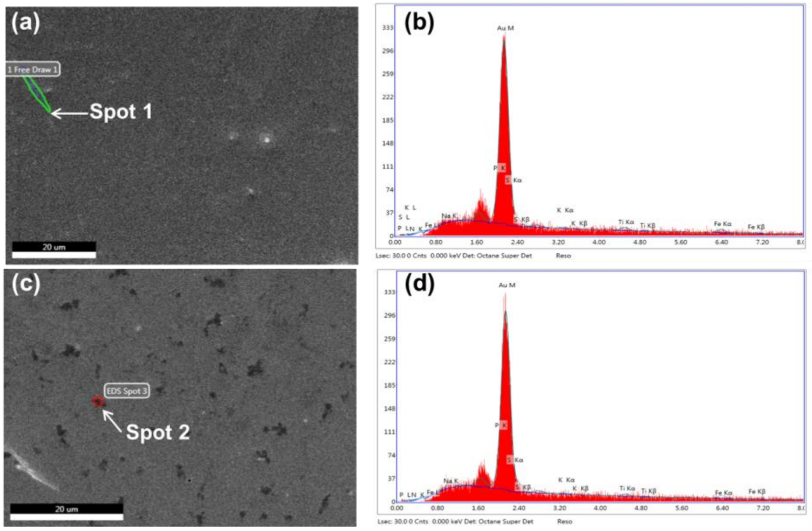

(e)	Bare electrode (Spot 1)	Regenerated electrode (Spot 2)
C K	0.18	0.19
N K	0.22	0.23
O K	0.08	0.09
NaK	13.15	12.83
P K	7.3	6.68
**AuM**	**57.13**	**58.74**
S K	0.02	0.02
K K	1.64	2.3
TiK	5.97	5.27
FeK	14.31	13.64

Figure S6. SEM images of fresh (a) and 25 times regenerated (c) microelectrode surface with spots (spot 1 and spot 2) indicated by arrows. EDX analysis of the spot1 (b) and spot 2 (d) with elemental content represented in table (e).

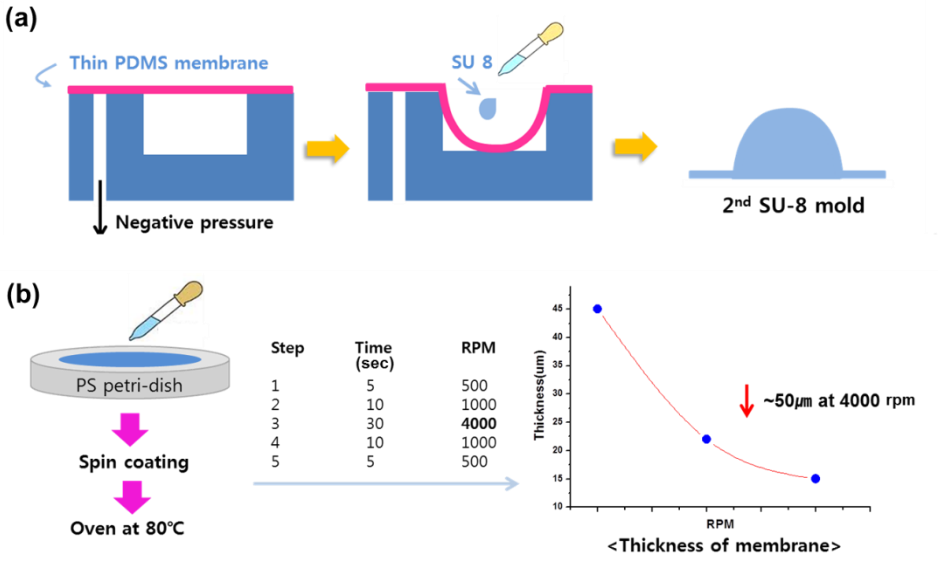

Figure S7. (a) Method of fabrication for 2nd SU-8 mold from the square microchannel using thin membrane and thin PDMS membrane. (b) Fabrication of thin PDMS membrane by spin coating at various RPM and thickness.

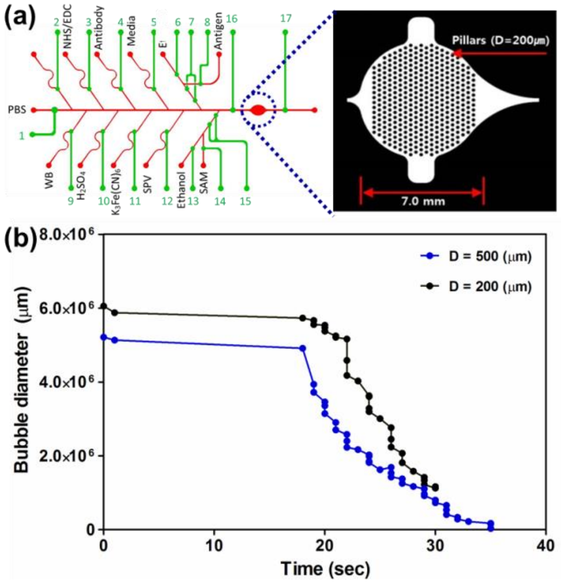

Figure S8. (a) The magnified image of a bubble trap in the microfluidic device. (b) Bubble removal from a microfluidic channel with time dependence according to the initial bubble diameter.

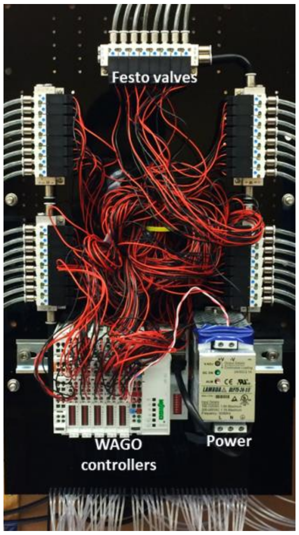

Figure S9. The microfluidic channel valve controller platform containing five sets of 8-channel pneumatic valves connected to WAGO controllers.

4
3
2
1
Bare electrode

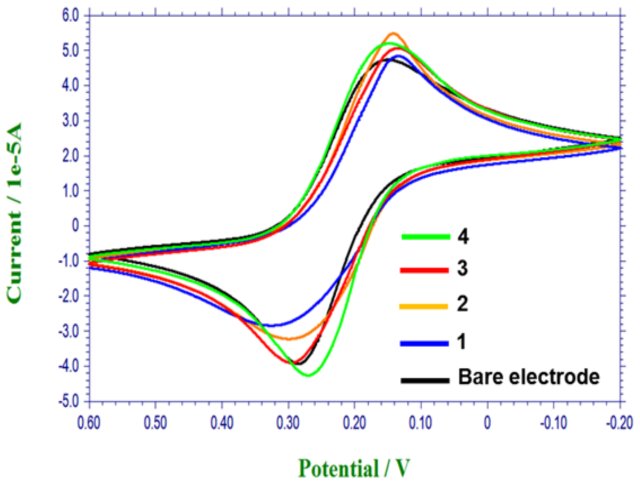

4
3
2
1
Bare electrode

No	Cleaning solution	Number of CV cycle	Flow rate (1000 μl/hr)	Recovered current rate (at 128mV)
1	10 mM H_2SO_4 /50 mM $K_3Fe(CN)_6$	3 cycles / 3 cycles	0	**53 %**
2	10 mM H_2SO_4 /50 mM $K_3Fe(CN)_6$	6 cycles / 3 cycles	0	**79 %**
3	10 mM H_2SO_4 /50 mM $K_3Fe(CN)_6$	5 cycles / 3 cycles	1000	**100 %**
4	10 mM H_2SO_4 /50 mM $K_3Fe(CN)_6$	6 cycles / 3 cycles	1000	**109 %**

Figure S10. On-chip electrode regeneration. CV measurements after the regeneration processes with different numbers of CV cycles and flow rates. Table showing the details of four sets of regeneration parameters and the resulting regeneration efficiencies.

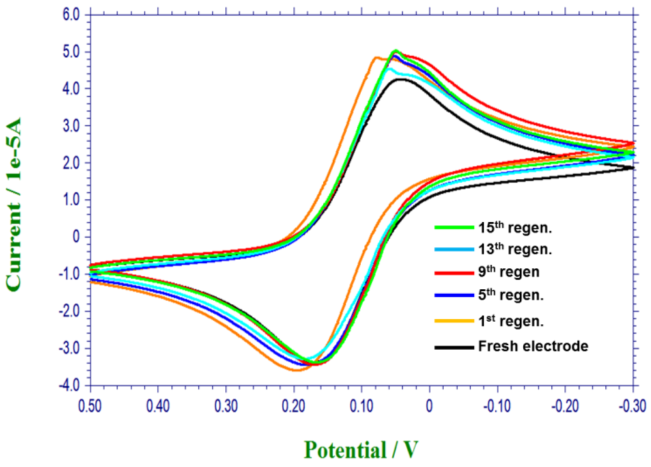

Figure S11. Comparison of CV scans after repeated regeneration of the microelectrodes.

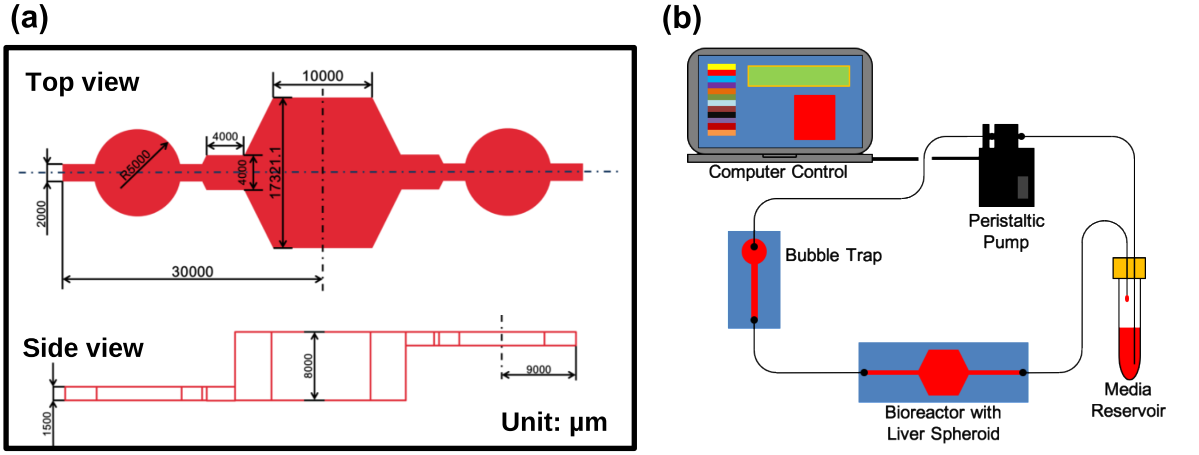

Figure S12. (a) Technical drawing of the liver bioreactor used throughout experiments. (b) The controlled system consists of peristaltic pump, liver bioreactor, media reservoir and computer control.

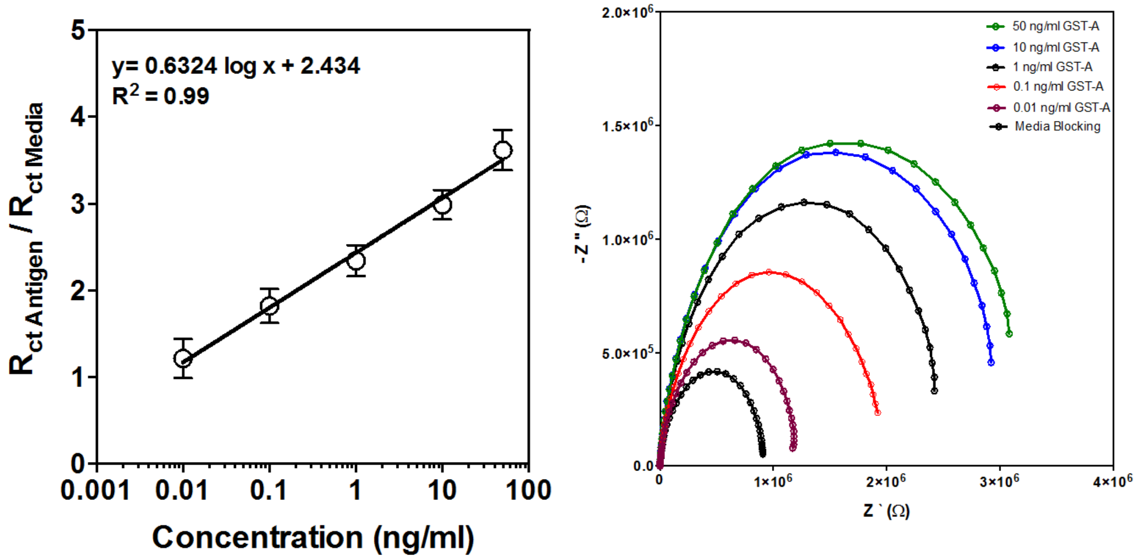

Figure S13. Calibration curve of GST-α drawn by normalized R_{ct} values ($R_{ct\text{-}Antigen}/R_{ct\text{-}Media}$) calculated according to the Randles circuit of nyquist plots ($R_s[R_{ct}C_{dl}W]$) constructed at different GST-α concentrations (0.01-50 ng/ml) (n=3).

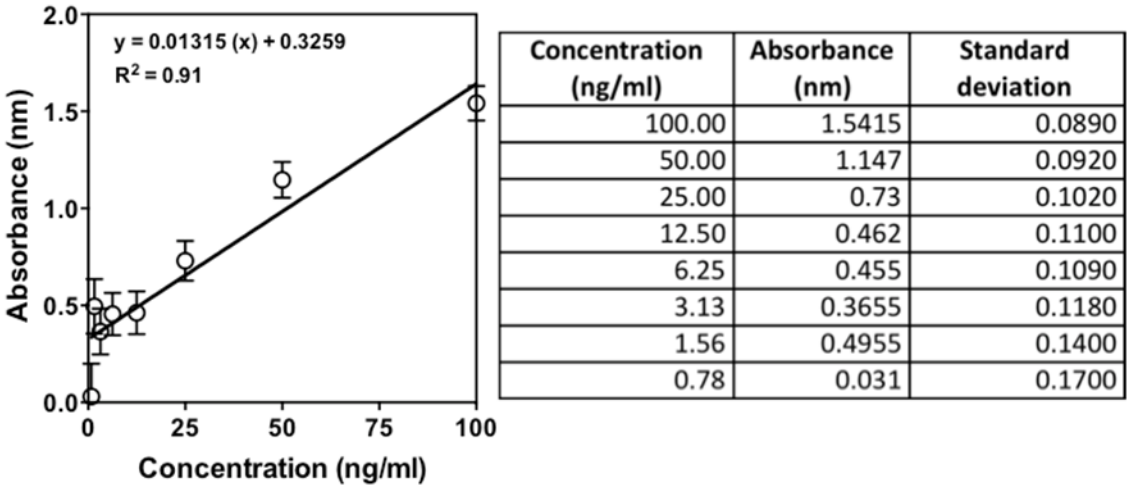

Concentration (ng/ml)	Absorbance (nm)	Standard deviation
100.00	1.5415	0.0890
50.00	1.147	0.0920
25.00	0.73	0.1020
12.50	0.462	0.1100
6.25	0.455	0.1090
3.13	0.3655	0.1180
1.56	0.4955	0.1400
0.78	0.031	0.1700

Figure S14. Calibration curve of albumin based on ELISA readings at 460 nm values of absorbance read at various albumin concentrations with standard deviation (n=3).

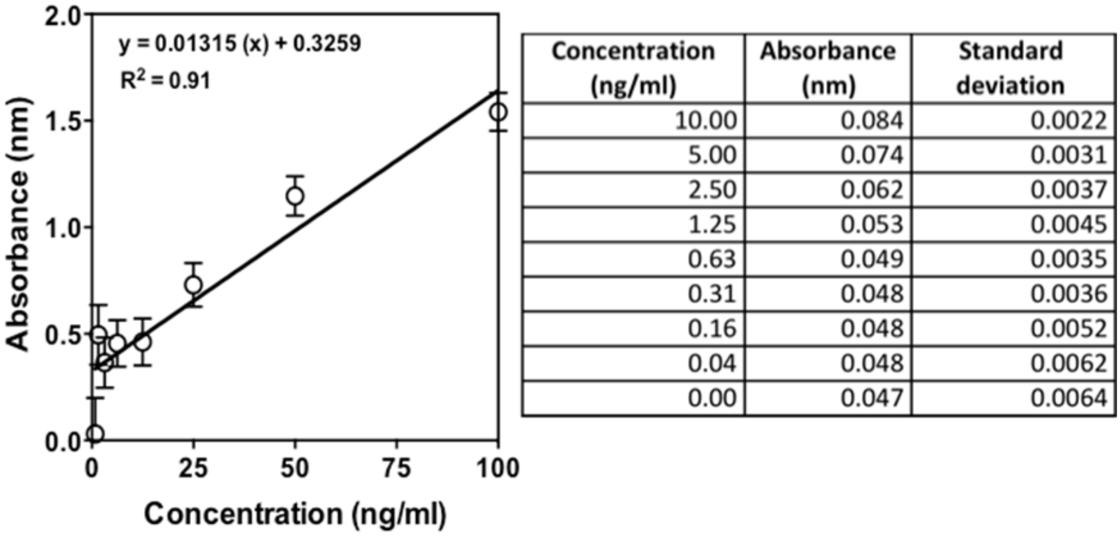

Concentration (ng/ml)	Absorbance (nm)	Standard deviation
10.00	0.084	0.0022
5.00	0.074	0.0031
2.50	0.062	0.0037
1.25	0.053	0.0045
0.63	0.049	0.0035
0.31	0.048	0.0036
0.16	0.048	0.0052
0.04	0.048	0.0062
0.00	0.047	0.0064

Figure S15. Calibration curve of GST-α based on ELISA readings at 460 nm values of absorbance read at various GST-α concentration with standard deviation (n=3).

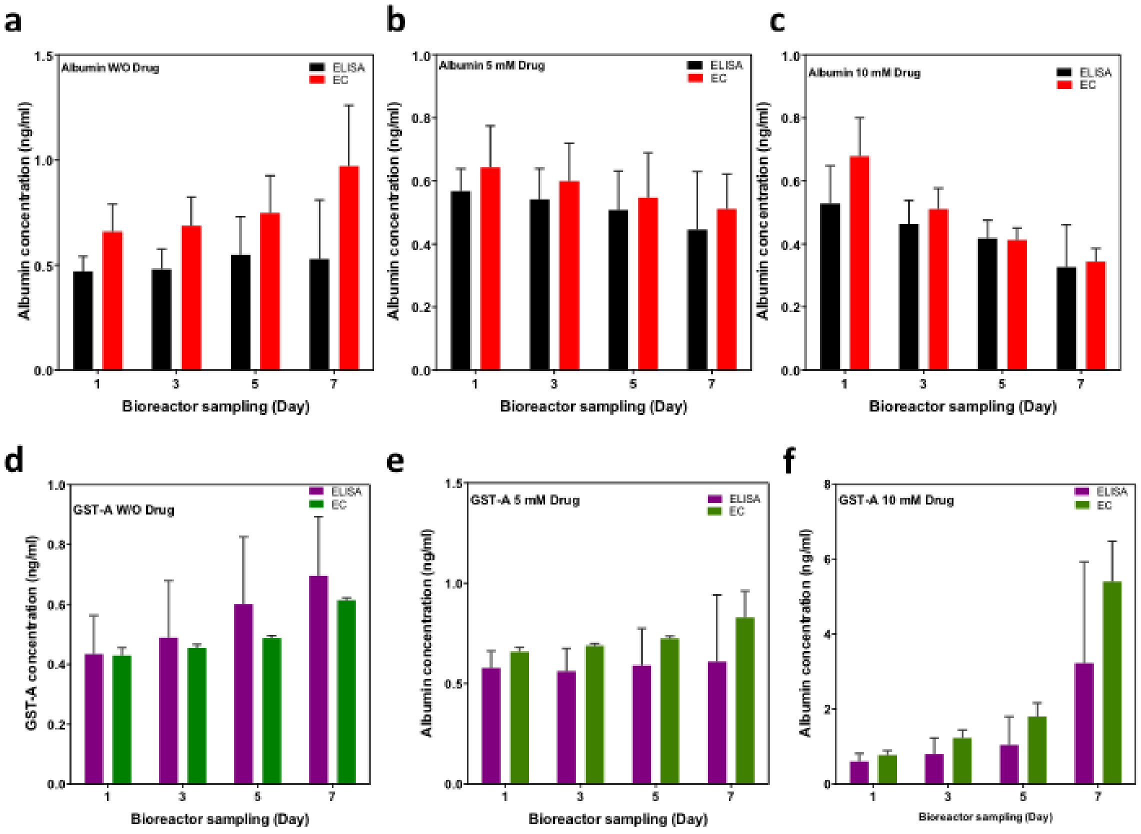

Figure S16. Bar graphs showing the change in concentration of albumin (a-c) and GST-α (d-f) for control, 5mM and 10 mM APAP exposed bioreactor samples (n=3).

References

[1] D. E. Souto, J. V. Silva, H. R. Martins, A. B. Reis, R. C. Luz, L. T. Kubota, F. S. Damos, *Biosens. Bioelectron.* **2013**, *46*, 22.

[2] C. Y. Li, K. R. Stevens, R. E. Schwartz, B. S. Alejandro, J. H. Huang, S. N. Bhatia, *Tissue Engineering Part A.* **2014**, *20*, 2200.

[3] L. E. Bertassoni, J. C. Cardoso, V. Manoharan, A. L. Cristino, N. S. Bhise, W. A. Araujo, P. Zorlutuna, N. E. Vrana, A. M. Ghaemmaghami, M. R. Dokmeci, *Biofabrication.* **2014**, *6*, 024105.

[4] a) N. S. Bhise, V. Manoharan, S. Massa, A. Tamayol, M. Ghaderi, M. Miscuglio, Q. Lang, Y. Shrike Zhang, S. R. Shin, G. Calzone, N. Annabi, T. D. Shupe, C. E. Bishop, A. Atala, M. R. Dokmeci, A. Khademhosseini, *Biofabrication.* **2016**, *8*, 014101; b) R. Riahi, S. A. Shaegh, M. Ghaderi, Y. S. Zhang, S. R. Shin, J. Aleman, S. Massa, D. Kim, M. R. Dokmeci, A. Khademhosseini, *Sci Rep.* **2016**, *6*, 24598.

[5] I. Ciani, H. Schulze, D. K. Corrigan, G. Henihan, G. Giraud, J. G. Terry, A. J. Walton, R. Pethig, P. Ghazal, J. Crain, *Biosens. Bioelectron.* **2012**, *31*, 413.
